# Rare functional variants in the *CRP* and *G6PC* genes modify the relationship between obesity and serum C‐reactive protein in white British population

**DOI:** 10.1002/mgg3.2255

**Published:** 2023-07-26

**Authors:** Xia Li, Alexander Ploner, Yunzhang Wang, Jonathan K. L. Mak, Yi Lu, Patrik K. E. Magnusson, Juulia Jylhävä, Sara Hägg

**Affiliations:** ^1^ School of Public Health and Emergency Management Southern University of Science and Technology Shenzhen China; ^2^ Shenzhen Key Laboratory of Cardiovascular Health and Precision Medicine Southern University of Science and Technology Shenzhen China; ^3^ Department of Medical Epidemiology and Biostatistics Karolinska Institutet Stockholm Sweden; ^4^ Social Sciences (Health Sciences) and Gerontology Research Center (GEREC) University of Tampere Tampere Finland

**Keywords:** C‐reactive protein, loss‐of‐function variants, obesity, protein‐altering variants, rare genetic variants, UK Biobank

## Abstract

**Background:**

C‐reactive protein (CRP) is a sensitive biomarker of inflammation with moderate heritability. The role of rare functional genetic variants in relation to serum CRP is understudied. We aimed to examine gene mutation burden of protein‐altering (PA) and loss‐of‐function (LOF) variants in association with serum CRP, and to further explore the clinical relevance.

**Methods:**

We included 161,430 unrelated participants of European ancestry from the UK Biobank. Of the rare (minor allele frequency <0.1%) and functional variants, 1,776,249 PA and 266,226 LOF variants were identified. Gene‐based burden tests, linear regressions, and logistic regressions were performed to identify the candidate mutations at the gene and variant levels, to estimate the potential interaction effect between the identified PA mutation and obesity, and to evaluate the relative risk of 16 CRP‐associated diseases.

**Results:**

At the gene level, PA mutation burdens of the *CRP* (*β* = −0.685, *p* = 2.87e‐28) and *G6PC* genes (*β* = 0.203, *p* = 1.50e‐06) were associated with reduced and increased serum CRP concentration, respectively. At the variant level, seven PA alleles in the *CRP* gene decreased serum CRP, of which the per‐allele effects were approximately three to seven times greater than that of a common variant in the same locus. The effects of obesity and central obesity on serum CRP concentration were smaller among the PA mutation carriers in the *CRP* (*p*_interaction_ = 0.008) and *G6PC* gene (*p*_interaction_ = 0.034) compared to the corresponding non‐carriers.

**Conclusion:**

PA mutation burdens in the *CRP* and *G6PC* genes are strongly associated with decreased serum CRP concentrations. As serum CRP and obesity are important predictors of cardiovascular risks in clinics, our observations suggest taking rare genetic factors into consideration might improve the delivery of precision medicine.

## Graphical abstract

The effects of obesity and central obesity on serum CRP concentration were smaller among the protein‐altering mutation carriers in the *CRP* (*p*_interaction_ = 0.008) and *G6PC* gene (*p*_interaction_ = 0.034) compared to the corresponding non‐carriers. As serum CRP and obesity are important predictors of cardiovascular risks in clinics, our observations suggest taking rare genetic factors into consideration might improve the delivery of precision medicine.

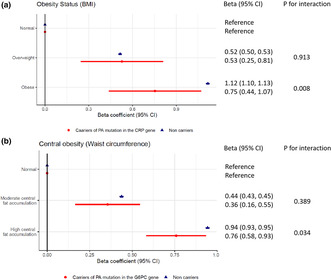

## 1 INTRODUCTION

C‐reactive protein (CRP) is a sensitive biomarker of inflammation and is routinely monitored in acute and chronic inflammatory conditions, including infections, inflammatory bowel diseases, autoimmune disorders, and cardiovascular diseases. Circulating CRP levels exhibit a drastic increase in the acute phase of an infection/injury, but often a constant and low‐grade increase in the presence of chronic inflammation. (Fu et al., 2020; Luan & Yao, 2018; Sproston & Ashworth, 2018) Serum concentration of CRP is found to be moderately heritable, with a twin‐based heritability ranging from 0.1 to 0.65. (Sas et al., 2017) Genome‐wide association studies (GWAS) and fine‐mapping studies have extensively scanned genetic variants across the human genome against serum CRP levels. (Kocarnik et al., 2018; Ligthart et al., 2018; Raffield et al., 2020; Said et al., 2022; Sinnott‐Armstrong et al., 2021) A recent meta‐GWASs found 266 independently associated loci, of which the lead SNPs together explained around 16.3% of the CRP variance, and revealed essential roles of immune and liver‐related metabolic pathways in CRP‐regulated mechanisms. (Ligthart et al., 2018; Said et al., 2022).

However, limited by statistical power, standard GWAS could only interrogate genetic variants that are common or of low frequency (minor allele frequency [MAF] >0.1%), even in the case of the UK Biobank (UKB) where genotypes were available for nearly half a million participants. (Sinnott‐Armstrong et al., 2021) In contrast to common genetic variants, rare variants are characterized by lower linkage disequilibrium (LD) with flanking variants, so that inferring causal loci is more straightforward than in a complex LD structure. (Momozawa & Mizukami, 2021) Besides, it is observed that the effects of rare variants can be potentially greater than the effects of common variants, (Park et al., 2011) making rare variants ideal instruments to identify biological mechanisms and prioritize therapeutic targets. Therefore, to complement our current understanding of genetic influences on CRP and to gain novel therapeutic insights, evidence from rare genetic variants is warranted.

Because of their low frequency, rare genetic variants are often grouped together by function before being subsequently associated with the trait of interest. (Lee et al., 2014; Povysil et al., 2019) A commonly used approach is the gene‐based burden test. Previously, Schick et al. performed an exome‐wide burden test for serum CRP among 6050 European Americans and 3109 African Americans and identified only one exome‐wide significant signal in the *CRP* gene (OMIM accession number: 123260). (Schick et al., 2015) Recently, researchers have analyzed the whole‐exome sequencing (WES) data from UKB. (Backman et al., 2021; Cirulli et al., 2020; Wang et al., 2021) Backman and colleagues used a large sample size with 454,787 UKB participants and identified two candidate genes associated with serum CRP in the burden test, the *CRP* and *G6PC* (glucose‐6‐phosphatase catalytic‐subunit; OMIM accession number: 613742) genes. (Backman et al., 2021) However, the previous UKB‐based studies investigated thousands of phenotypes simultaneously and there is a lack of follow‐up analyses focusing on serum CRP genetics to explore the clinical relevance of the identified genes in‐depth. Overall, our understanding of the rare functional mutations on serum CRP has been limited.

Besides genetic factors, obesity is another well‐established risk factor for chronic inflammation, with higher serum CRP concentrations being a marker of elevated adiposity. (Timpson et al., 2011) Previous studies using common genetic variants have observed that genetic predisposition to higher serum CRP modified the obesity–CRP relationship among both European and Asian populations. (Curocichin et al., 2011; Dehghan et al., 2011; Eiriksdottir et al., 2009; Teng et al., 2009) Both serum CRP level and obesity play an important role in the risk assessment of cardiovascular diseases, therefore exploring the effect modification due to genetic factors and obesity could guide a personalized risk prediction. However, there is a lack of evidence for rare genetic mutations in this regard. Moreover, the association between rare functional variants and disease risks could further facilitate the delivery of precision medicine, yet are still unknown.

Consequently, we aimed to examine the whole exome‐wide mutation burden due to rare coding variants that could lead to functional consequences, including protein‐altering (PA) and loss‐of‐function (LOF) mutations at both gene and variant levels, in association with serum CRP concentrations among 161,430 UKB participants with European ancestral background. Furthermore, we aimed to elucidate the clinical relevance of the identified genetic factors by estimating the gene–obesity interaction as well as the relative risk for a set of CRP‐associated diseases.

## 2 MATERIALS AND METHODS

### 2.1 Ethical compliance

UK Biobank had obtained ethics approval from the North West Multicentre Research Ethics Committee (11/NW/0382 and 16/NW/0274) and informed consent from all participants, which covered the approved use of the data in the present analyses (Approved Research ID: 22224).

### 2.2 Study population

The UKB is a large‐scale prospective cohort study and recruited >500,000 participants aged 40–69 years from across the United Kingdom. The initial assessment visit was conducted during 2006–2010 and is referred to as the baseline assessment in the present study. (Sudlow et al., 2015) Participants are extensively phenotyped and genotyped through various measurements, including questionnaire surveys, physical measures, bio‐sample assays, genome‐wide array genotyping with imputation, and whole‐exome sequencing (WES). In addition, diagnoses and death for all participants are followed up via hospital inpatient data and mortality data.

Data extraction for the present analysis is summarized in Figure S1; briefly, we included UKB participants whose (1) serum CRP measurement (Field 30,710) is available at baseline, (2) WES data are made available in the second WES 200 k release, and (3) self‐reported ethnic backgrounds (Field 21,000) are “white”. We then applied the following exclusion criteria: (1) who withdrew from the UKB study, (2) whose genotype‐inferred sex (Field 22,001) is different from the self‐reported sex (Field 31), (3) ancestral outliers, defined by a >3 standard deviations (SD) from the mean of genomic principal components (PCs) derived from the 1000 Genomes reference panel (phase 3 EUR) and the projected genomic PCs using arrayed genotype data in UKB, (4) who failed sample quality control (QC) for WES, namely had a >10% sample‐wise missing rate across all WES variants, (5) who failed sample QC for arrayed genotypes, including those showing extreme heterozygosity and missing rate across a set of high‐quality markers (Field 22,027), putatively carrying sex chromosome aneuploidy, and missing genomic PCs (UKB data showcase Resource 531 Sample‐QC). The inclusion and exclusion procedures above resulted in an eligible sample set with 177,242 individuals (i.e., the eligible set in Figure S1).

Next, we defined unrelated family clusters by grouping members with a third‐degree or higher relatedness (kinship coefficient > 0.0442) to the same family cluster. For each family cluster, we retained one random family member such that all the study participants are unrelated in the analysis (*N* = 15,812 were excluded; Figure S1). For the purpose of the whole exome‐wide gene burden test, we further separated the analysis set into two groups randomly, with each containing 75% and 25% of the unrelated participants and treated as the discovery and replication set, respectively. The combination of discovery and replication samples yielded a total of 161,430 participants and is referred to as the “meta‐analysis set” throughout (Figure S1).

### 2.3 Genotypes

#### 2.3.1 WES genotypes

WES genotypes were made available for 200,643 UKB participants in October 2020 (interim 200 k release, Field 23,155–23,156), of which the sequencing procedure and computation pipelines have been detailed elsewhere. (Van Hout et al., 2020) The genetic variants of primary interest were selected from the WES autosomal genotypes. We first performed variant‐wise QC by including (1) sites with mean read depth ≥ 10, (2) sites with missing rates ≤0.1, and (3) variants with genotype quality scores ≥20. Next, we restricted our analysis to coding sequence (CDS) regions and rare variants, namely MAF <0.1% in the present study population and in the gnomAD Non‐Finnish European population if the variant exists in the gnomAD exomes database (Karczewski et al., 2020). We further annotated the variants and predicted the functional consequences using Ensembl Variant Effect Predictor (VEP 103.1), (McLaren et al., 2016) Ensembl Homo sapiens reference database (103_GRCh38), and the Ensembl canonical transcripts.

Specifically, two types of variants, PA and LOF variants, are the focus of the present study. PA variants are single‐nucleotide variants (SNVs) or indels that lead to any of the following functional consequences: splice acceptor variant, splice donor variant, stop gained, frameshift, stop lost, start lost, inframe insertion, inframe deletion, and missense variant. To select deleterious variants, we used two pathogenicity scores, SIFT (Sorting Intolerant From Tolerant) (Ng & Henikoff, 2003) and PolyPhen (Polymorphism Phenotyping), (Adzhubei et al., 2010) to filter out benign missense variants (SIFT > 0.05 and PolyPhen < 0.15). Of all PA variants, a subset with high‐impact consequences, including splice acceptor variant, splice donor variant, stop gained, frameshift, stop lost, and start lost, are classified as LOF variants. As a result, 1,776,249 PA and 266,226 LOF variants contributed to the present analysis (Figure S2).

To perform the whole exome‐wide gene‐based burden tests, we subsequently collapsed the PA variants according to the mapped gene regions (Ensembl Human genes [GRCh38.p13]). That is, for each gene, participants were assigned to value 1 if carrying at least one PA allele in the corresponding gene region, i.e., mutation carriers, and to value 0 otherwise, i.e., non‐carriers. We excluded the genes without PA mutation carriers, i.e., no genotype variant observed among the present study population, and analysed 21,270 genes in the PA mutation burden test. Similarly, LOF variants were collapsed into 20,047 genes in the LOF mutation burden test.

#### 2.3.2 Imputed genotypes and genomic PCs

Genome‐wide arrayed and imputed genotypes are available for about 488,000 UKB participants and a detailed measurement procedure was described previously. (Bycroft et al., 2018) As a comparison for rare mutations, we also explored a known strong CRP‐associated common genetic variant, rs1205 (C>T, allele frequency of T: 0.33) located at the *CRP* gene on chromosome 1, from the imputed genotype data. In addition, we calculated the polygenetic risk score of CRP by summing the CRP‐increasing alleles over 58 SNPs weighted by their effects on serum CRP. The SNP list and corresponding weights were determined from a previous meta‐GWAS. (Said et al., 2022).

Genomic PCs in UKB were created from a representative set of LD‐pruned, high‐quality common variants using arrayed genotypes. The methods of PC calculation were described elsewhere (UKB document: Genotyping and quality control of UK Biobank, a large‐scale, extensively phenotyped prospective resource) and the individual‐level data of PCs were directly downloaded from the UKB data showcase (Resource 531 Sample‐QC file).

### 2.4 Phenotypes

#### 2.4.1 CRP

CRP (Field 30,710) was measured by the high‐sensitivity immunoturbidimetric assay. CRP level at baseline assessment was analysed in the present study. The original value of CRP (in mg/L) presented a skewed distribution. Therefore, the natural logarithm of CRP was calculated and used in the statistical analyses throughout.

#### 2.4.2 Obesity

We used two measures, BMI (unit: kg/m^2^, field 23,104 and 21,001) and waist circumference (unit: cm, field 48), to define the obese and central obese status, respectively. Continuous BMI values fall into four categories: underweight (<18.5 kg/m^2^), normal (18.5–24.9 kg/m^2^), overweight (25.0–29.9 kg/m^2^), and obese (≥30.0 kg/m^2^). Indexed by sex‐specific waist circumference, the three central obesity groups were normal fat distribution (women <80 cm or men <94 cm), moderate central fat accumulation (80 cm ≤women <88 cm or 94 cm ≤men <102 cm), and high central fat accumulation (women ≥88 cm or men ≥102 cm) (Han & Lean, 2016).

#### 2.4.3 CRP‐associated diseases

We examined a list of CRP‐associated diseases in the present study. The disease list was primarily selected from a previous study (Prins et al., 2016). We excluded diseases for which zero cases were observed among the carriers of PA mutations of interest identified in the present burden test, such that a relative risk can be estimated by contrasting the risk among carriers with non‐carriers. The final CRP‐associated disease list comprises autoimmune and inflammatory (celiac disease, all types of inflammatory bowel disease [IBD], Crohn's disease, ulcerative colitis, psoriatic arthritis, rheumatoid arthritis, type 1 diabetes, knee osteoarthritis), cardiovascular (coronary artery disease, ischaemic stroke), metabolic (type 2 diabetes, chronic kidney disease), neurodegenerative (Alzheimer's disease, Parkinson's disease) diseases, and psychiatric (bipolar disorder, depressive disorder) disorders.

We used three types of data sources to ascertain the occurrence of a disease diagnosis, namely self‐reported health conditions, hospital inpatient data, and causes of death. Table S1 lists relevant values and ICD codes used in the disease ascertainment. All participants reported their prevalent diseases (Field 20,002) and a recollected diagnostic date through a questionnaire survey at baseline assessment. In addition, we used International Classification of Diseases (ICD) codes to determine the presence of a diagnosis in the primary and secondary causes of the hospital inpatient episodes (ICD9 and ICD10) and of the death records (ICD 10). Using hospital inpatient episodes, the date of diagnosis was proxied by the first non‐missing value across the following dates: episode start, admission to hospital, episode end, and discharge from hospital. When the diagnosis was detected as the cause of death, the death date was assigned to the diagnostic date. We treated a diagnosis occurrence captured by any source as the presence of a disease and the first occurrence date was defined as the earliest diagnostic date across all occurrences. Self‐reported diagnoses were collected at the UKB baseline assessment; inpatient data and death information are updated through June 2021.

#### 2.4.4 Other variables

We also described the distributions of a number of demographic, socioeconomic, behavioural, and anthropometric factors, which are common factors of serum CRP, measured at the baseline assessment, including age (Field 21,003), sex (Field 31), education, physical activity, ever smoked (Field 20,160), daily alcohol intake, systolic blood pressure (Field 93 and 4080), glucose (Field 30,740), triglycerides (Field 30,870), and high‐density lipoprotein (HDL) cholesterol (Field 30,760). Years of birth (Field 34) were binned in a 10‐year interval to indicate the cohort effect. Education, physical activity, and daily alcohol intake were self‐reported and were derived from the questions of “qualification” (Field 6138), “types of physical activity” (Field 6164), and alcohol intake frequency (Field 1558), respectively. Three education levels include high (college or university degree), intermediate (A levels, O levels/GCSEs, CSEs, NVQ/HND/HNC, other professional qualifications), and low (no relevant qualifications); four categories of physical activity are none (none), low (light do‐it‐yourself [DIY]), medium (walking for pleasure, or other exercises), and high (heavy DIY and strenuous sports); daily alcohol intake is defined as those who reported “daily or almost daily” for alcohol intake frequency. In addition, the release of WES data was treated as a dichotomized covariate in the WES analyses throughout because the samples in the initial 50 k release and the remaining samples in the second 200 k release were sequenced on different flow cell platforms.

#### 2.4.5 Statistical analysis

We first described the characteristics of the study population at baseline and the features of the genetic variants and genes included in the present analysis. Variant‐wise, we summarized the number and proportion of variants by functional consequence types, as well as by MAF categories; gene‐wise, we described the gene length, the number of PA/LOF variants per gene, and the number of mutation carriers per gene.

#### 2.4.6 PA mutation–CRP associations at the gene level

Next, we performed whole exome‐wide burden tests by aggregating PA variants into gene‐based burden scores and associated those with serum CRP concentration. We adopted a Combined Multivariate and Collapsing (CMC) method, (Li & Leal, 2008) meaning that for the mutation burden of each gene, we assigned 1 to individuals carrying at least one PA/LOF allele within the corresponding gene region, and 0 to individuals otherwise. The effect sizes (*β* coefficients and standard errors [SE]) measure the effect of the gene burden due to the presence of any PA/LOF allele on natural log‐transformed CRP levels, with a positive value indicating a CRP‐increasing effect. We performed the CMC burden test in both the discovery set and the replication set. The whole exome‐wide significance levels for PA and LOF mutation burden were set to 2.35e‐06 (=0.05/21,270) and 2.49e‐06 (=0.05/20,047), respectively. Genes that reached the whole exome‐wide significance level in the Discovery set and a <0.05 *p* value in the Replication set were considered significant. Two sets of covariates were considered in the burden tests, of which the first model controlled for age, sex, WES release information, and 20 genomic PCs (Model 1), and the second model additionally adjusted for BMI on top of the first model (Model 2). All burden tests were fitted in Rvtests (version: 20190205). (Zhan et al., 2016).

Using the summary statistics derived from the discovery set, we further examined genomic inflation by comparing the observed statistics against the expected statistics under the null hypothesis of no association. Results of burden tests and genomic inflations were visualized in Manhattan plots and quantile–quantile (Q–Q) plots, respectively. LOF variants were examined in a similar manner.

To examine whether the effects of PA mutations were independent of the common genetic variants, we next fitted secondary models with additional adjustment for the polygenic risk score (PRS) of CRP, which is derived from 58 common variants. In addition to the CMC burden test, we also conducted sensitivity analyses by using two alternative gene‐based methods in the discovery set. The first one is the Morris‐Zeggini (MZ) burden test, (Morris & Zeggini, 2010) where the number of rare PA alleles within the gene was treated as the value of gene burden. The second method is the optimal sequence kernel association (SKAT‐O) test, (Lee et al., 2012) which is a combined burden and variance‐component test and is more powerful in the presence of both trait‐increasing and trait‐decreasing variants. To minimize a possible confounding effect due to acute inflammation, we did a third sensitivity analysis by excluding individuals whose baseline CRP values were > 10 mg/L, and re‐estimating the CMC burden tests in the discovery set.

#### 2.4.7 PA mutation–CRP associations at the variant level

Once candidate genes from the above burden tests were identified, a closer inspection at the PA‐variant level was performed. First, we estimated the single PA variant–CRP association. Model covariates were the same as in the burden test. Variants with false discovery rate (FDR) ≤0.05 were considered candidate variants. Second, to contrast the per‐allele effects of rare variants with that of a common variant, we also estimated the effect of the rs1205 (C>T, frequency of T allele =33.0% in the Meta‐analysis set), a top signal in the *CRP* gene. Third, we performed “leave‐one‐out” variant analyses to check the influence of every single variant on the overall gene burden effect by re‐estimating the burden test with the exclusion of one variant from the total PA variant list at a time.

#### 2.4.8 Interaction effect between PA mutation burden and obesity categories

We examined the potential interplay between PA mutation burden (genetic exposure) in the significant genes and the obesity and central obesity categories (environmental exposure), grouped by BMI and waist circumference respectively, in controlling serum CRP level (outcome). Two types of models were fitted: (1) serum CRP being a function of obesity status in PA mutation carriers and non‐carriers, respectively (stratified models), where the PA status‐specific effects of the BMI and waist circumference categories on serum CRP were estimated; and (2) serum CRP being a function of PA mutation, obesity status, and a multiplicative term between PA status and obesity categories (interaction models), where the *p* value for the multiplicative term was reported as the *p* value for interaction. All models were adjusted for age, sex, birth year category, WES release, and 20 genomic PCs.

#### 2.4.9 PA mutation burden and CRP‐associated diseases

Finally, we estimated the associations between PA mutation burden and the risks of CRP‐associated diseases (listed in the “Method‐CRP‐associated diseases” section previously). Logistic regression models were fitted to estimate effect sizes (odds ratios [OR]). For each disease, we fitted a model adjusting for age, sex, birth year category, WES release, and 20 genomic PCs. FDR was used to correct multiple testing.

An overview of the study aims and analysis plan is illustrated in Figure S3. Data management was performed using vcftools 0.1.16, (Danecek et al., 2011) PLINK 2.0, (Chang et al., 2015) and R 4.0.5; all statistical models were fitted in Rvtests (version: 20190205) (Zhan et al., 2016) and R 4.0.5; data visualizations were completed in R 4.0.5 with functions loaded from the packages of “qqman”, “trackViewer”, and “ggplot2”.

## 3 RESULTS

### 3.1 Baseline characteristics of the study population and the genetic variants

A total of 161,430 eligible and unrelated participants contributed to the present analysis (meta‐analysis set). All participants, of whom 45.1% were men, had an average age of 56.7 years, a mean BMI of 27.3 kg/m^2^, and a median serum CRP of 1.30 mg/L. The baseline characteristics of the discovery set were not appreciably different from the replication set (Table 1).

**TABLE 1:** Baseline characteristics of the study population.

	Discovery set	Replication set	Meta‐analysis set	*p* ^a^
Number of participants	121,072	40,358	161,430	
Age (year)^b^	56.7 (8.0)	56.6 (8.0)	56.7 (8.0)	0.21
Men^c^	54,583 (45.1%)	18,182 (45.1%)	72,765 (45.1%)	0.92
High physical activity^c^	58,672 (48.6%)	19,400 (48.3%)	78,072 (48.5%)	0.18
Ever‐smoker^c^	74,363 (61.6%)	24,825 (61.7%)	99,188 (61.7%)	0.72
Daily alcohol user^c^	25,773 (21.3%)	8486 (21.0%)	34,259 (21.2%)	0.27
BMI (kg/m^2^)^b^	27.3 (4.7)	27.3 (4.7)	27.3 (4.7)	0.88
Waist circumference (cm)^b^	90.0 (13.4)	90.0 (13.4)	90.0 (13.4)	0.89
Systolic blood pressure (mm Hg)^b^	137.8 (18.5)	137.9 (18.5)	137.8 (18.5)	0.28
Serum biomarkers				
Glucose (mmol/L)^b^	5.11 (1.18)	5.11 (1.18)	5.11 (1.18)	0.56
Triglycerides (mmol/L)^d^	1.48 (1.09)	1.48 (1.09)	1.48 (1.09)	0.90
HDL cholesterol (mmol/L)^b^	1.46 (0.38)	1.46 (0.38)	1.46 (0.38)	0.58
CRP (mg/L)^d^	1.30 (2.03)	1.30 (2.05)	1.30 (2.03)	0.86
Natural logarithm CRP^b^	0.31 (1.05)	0.31 (1.06)	0.31 (1.06)	0.72

Abbreviations: BMI, body mass index; CRP C‐reactive protein; HDL, high‐density lipoprotein.

^a^ *p* for group difference between the discovery set and the replication set, estimated by Fisher's exact test, *t*‐test, and non‐parametric median test, as appropriate.

^b^ Statistics—mean (standard deviation).

^c^ Statistics—number of participants (proportion).

^d^ Statistics—median (interquartile range).

The present study analysed 1,776,249 PA and 266,226 LOF variants, of which missense variants (81.7%) and frameshift variants (53.9%) were the most common types, respectively (Table S2). The MAFs of 70.2% PA and 75.9% LOF variants fell into the category of 0.0001%–0.001%. A total of 21,270 PA‐harbouring genes were studied, covering a median number of 71 PA variants/gene; the corresponding number was 12 LOF variants/gene for 20,047 LOF‐harbouring genes (Table S3).

### 3.2 PA mutation–CRP associations at the gene level

**FIGURE 1:**
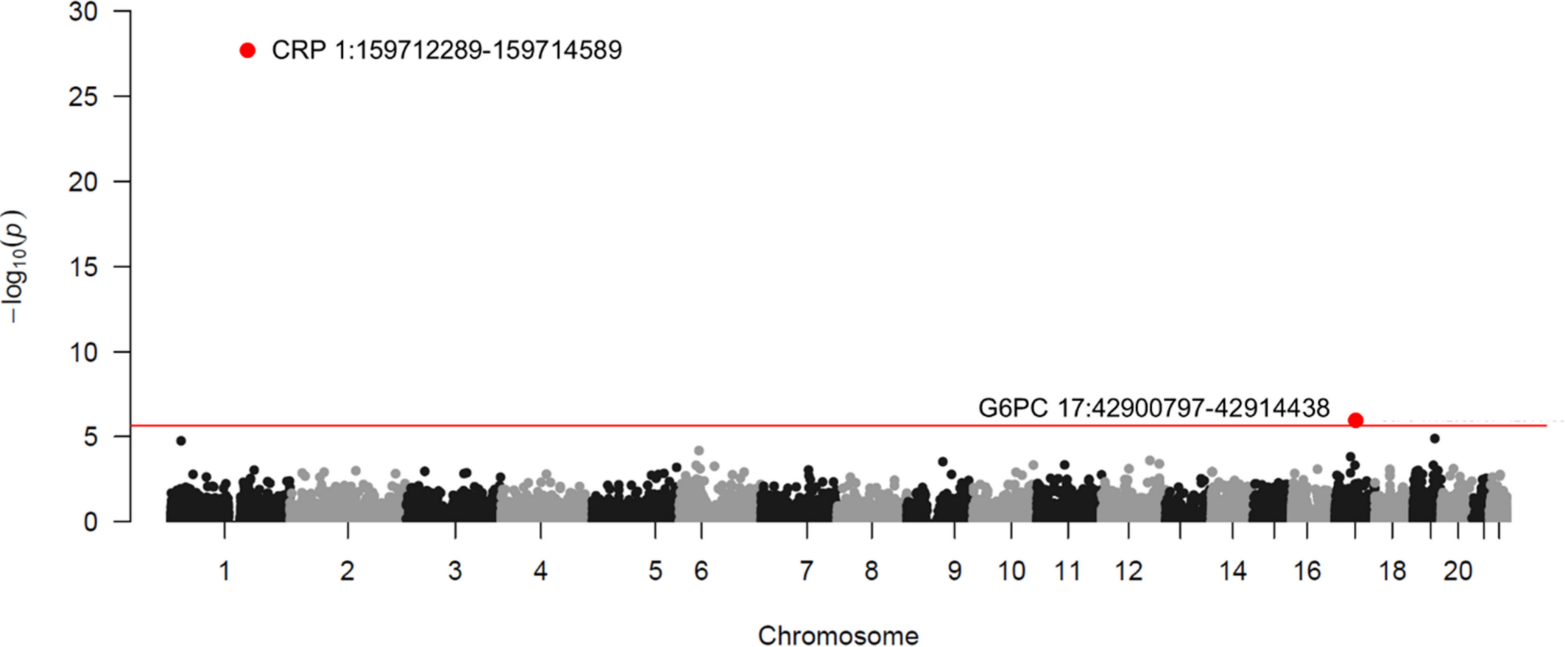
Associations between gene‐based PA mutation burden and serum CRP in the discovery set.

Using the CMC gene‐based burden test and a whole exome‐wide significant level (*p* < 2.35e‐06), we found that PA mutation burden in the *CRP* and *G6PC* genes was significantly associated with serum CRP concentration (Figure 1; Table 2). Specifically, we controlled two sets of covariates in the statistical models and the *G6PC* gene reached significance only in Model 2, where BMI was additionally adjusted for on top of Model 1 (Table S4; Figure S4). Genes, other than the *CRP* and *G6PC* genes, did not show significant effects (Figure 1; Figure S4).

**TABLE 2:** Whole exome‐wide significant associations between PA/LOF mutation burden and natural log‐transformed serum CRP concentration.

Mutation and population	Number of individuals	No. of PA/LOF variants	No. of carriers of PA/LOF alleles	*β* (SE)	*p* value
PA mutation in the *CRP* gene					
Discovery	120,687	52	229	−0.685 (0.062)	2.87e‐28
Replication	40,231	52	83	−0.704 (0.103)	9.10e‐12
PA mutation in the *G6PC* gene					
Discovery	120,687	76	496	0.203 (0.042)	1.50e‐06
Replication	40,231	76	163	0.253 (0.074)	6.02e‐04
LOF mutation in the *CRP* gene					
Discovery	120,687	12	63	−0.753 (0.118)	1.96e‐10
Replication	40,231	12	25	−0.825 (0.188)	1.13e‐05

*Note*: Beta regression coefficient of the PA mutation burden, SE standard error. Age, sex, WES release information, 20 genomic PCs, and BMI were controlled in the statistical models (Model 2). Of the study population, 512 individuals had missing BMI at baseline; therefore, the numbers of individuals were slightly different from those in Table S4. *CRP* gene: Chromosome 1 ‐ NC_000001.11 using assembly GRCh38.p14; *G6PC* gene: Chromosome 11 ‐ NC_000077.7 using assembly GRCh38.p14.

Carrying any PA allele in the *CRP* gene decreased the natural log‐transformed CRP by 0.685 (equivalent to a 49.6% reduction on mg/L scale; *p* = 2.87e‐28 in the discovery set and *p* = 9.10e‐12 in the replication set); on the contrary, PA mutation carriers in the *G6PC* gene showed a 0.203 increase in the log serum CRP (equivalent to a 22.5% increase on mg/L scale; *p* = 1.50e‐06 in the discovery set and *p* = 6.02e‐04 in the replication set; Table 2). A Q‐Q plot did not suggest obvious evidence of an inflation concern (Figure S5). The burden test for the LOF mutation demonstrated that the *CRP* locus was the only significant signal across the autosomal exome (*β* = −0.753 [equivalent to a 52.9% reduction on mg/L scale], *p* value = 1.96e‐10 in the discovery set and *p* = 1.13e‐05 in the replication set; Table 2 and Table S4).

Particularly, the genetic effects of PA mutation burden in the *CRP* and *G6PC* genes largely remained the same after adjusting for the PRS of serum CRP, suggesting genetic effects of rare mutations were independent of the common variants (Table S5). Sensitivity analyses, including using the MZ burden test, conducting the SKAT‐O analysis, and excluding the participants with >10 mg/L CRP at baseline from the study population, did not reveal additional signals (Figures S6–S8).

The Manhattan plot shows the associations between gene‐based PA mutation burden and serum CRP (natural‐log transformed), estimated by CMC burden test in the discovery set. Age, sex, WES release information, 20 genomic PCs, and BMI were controlled in the statistical models (Model 2). Each dot represents a gene, of which the location is determined by the chromosome and position on X scale and the statistic value of –log10(*p*) on Y scale. The red horizontal line denotes the whole exome‐wide significant level (*p* < 2.35e‐06). Annotations, including gene name, chromosome, and location, of the exome‐wide significant genes were displayed in the plot. *CRP* gene: Chromosome 1 ‐ NC_000001.11 using assembly GRCh38.p14; *G6PC* gene: Chromosome 11 ‐ NC_000077.7 using assembly GRCh38.p14.

### 3.3 PA mutation–CRP associations at the variant level

Next, we examined the PA mutation–CRP association at the variant level across the candidate loci, i.e., the *CRP* and *G6PC* genes, using the Meta‐analysis set. A total of 52 PA variants, of which 12 were LOF variants, were analysed in the coding region of the *CRP* gene; the corresponding numbers for the *G6PC* gene were 76 PA variants and 16 LOF variants. In the present study, with respect to any PA and LOF variants in the *CRP* and *G6PC* genes, the mutation carriers were heterozygous (Table S6).

**FIGURE 2:**
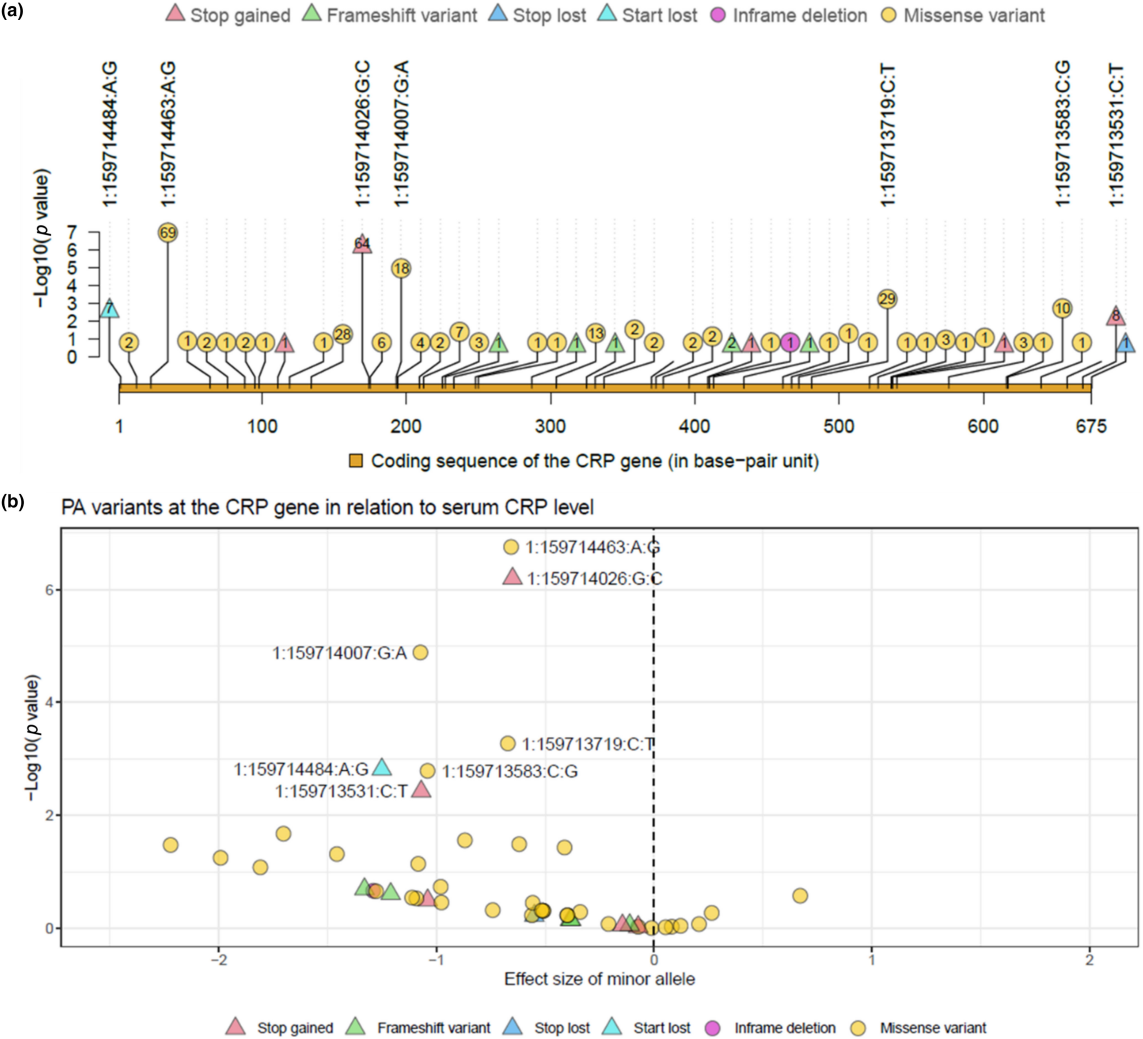
PA variants in the coding sequence of the *CRP* gene and the associations with serum CRP in the meta‐analysis set. (a) The plot shows the location of PA variants in the coding sequence region of the *CRP* gene on X scale and the value of –log10(*p*) on Y scale. LOF variants were displayed in triangles, while the remaining PA variants were displayed in circles, which contain the number of mutation carriers. Consequence types were denoted by different colours. Variant IDs of the FDR significant variants were displayed in the plot. (b) The volcano plot illustrates the effect sizes of PA variants on X scale and the value of –log10(*p*) in Y scale. Shape, colour, and text annotations were the same as those in Panel (a). *CRP* gene: Chromosome 1 ‐ NC_000001.11 using assembly GRCh38.p14.

Of the PA alleles in the *CRP* gene, a majority (*n* = 43) exhibited CRP‐decreasing effects (Figure 2b; Table S6). Seven PA variants (including four missense, two stop gained, and one start loss variants) were associated with serum CRP at an FDR significance level, and all of them showed decreasing effect (Figure 2; Table 3). Of the FDR significant variants, the smallest *p* value was observed for a missense variant, 1:159714463:A:G, with 69 heterozygous mutation carriers expressing a 0.66 lower level in the log serum CRP (equivalent to a 48% reduction on mg/L scale) compared to the non‐carriers; a start loss mutation, 1:159714484:A:G, showed the largest effect size and seven heterozygous mutation carriers on average showed a 1.25 reduction in the log serum CRP (equivalent to a 71% reduction on mg/L scale). The per‐allele effect across the seven FDR significant PA variants ranged from −0.65 to −1.25, approximately three to seven times greater than that of rs1205‐C>T, a common variant and a strong signal in the *CRP* gene, of which the per‐T‐allele effect was −0.175 (Table S7).

**TABLE 3:** The associations between FDR significant PA variants in the *CRP* gene and serum CRP (natural‐log transformed) in the meta‐analysis set.

Variant ID	Genomic placement	Consequense type	CDS position	Amino acids	Number of individuals	Alternate allele frequency	*β* (SE)	*p* value
1:159714463:A:G	NC_000001.11:g.159714463A>G	Missense	23	L/S	161,361/69/0	2.14e‐04	−0.657 (0.126)	1.76e‐07
1:159714026:G:C	NC_000001.11:g.159714026G>C	Stop gained	174	Y/*	161,366/64/0	1.98e‐04	−0.65 (0.131)	6.40e‐07
1:159714007:G:A	NC_000001.11:g.159714007G>A	Missense	193	R/C	161,412/18/0	5.58e‐05	−1.074 (0.246)	1.30e‐05
1:159713719:C:T	NC_000001.11:g.159713719C>T	Missense	481	G/S	161,401/29/0	8.98e‐05	−0.672 (0.194)	5.33e‐04
1:159714484:A:G	NC_000001.11:g.159714484A>G	Start lost	2	M/T	161,423/7/0	2.17e‐05	−1.251 (0.395)	1.54e‐03
1:159713583:C:G	NC_000001.11:g.159713583C>G	Missense	617	R/P	161,420/10/0	3.10e‐05	−1.041 (0.33)	1.63e‐03
1:159713531:C:T	NC_000001.11:g.159713531C>T	Stop gained	669	W/*	161,422/8/0	2.48e‐05	−1.07 (0.369)	3.77e‐03

*Note*: Variant ID is in Chromosome: position: reference allele: alternate allele format; Genomic placement: GRCh38.p14 chromosome 1 used as reference subsequence; CDS position: relative position of base pair in coding sequence; Amino acids: Reference/variant amino acids, R Arginine, C Cysteine, G Glycine, L Leucine, M Methionine, P Proline, S Serine, T Threonine, W Tryptophan, Y Tyrosine; Number of individuals: number of reference allele homozygote, heterozygote, and alternate allele homozygote; Beta: regression coefficient of the alternate allele, SE standard error. *CRP* gene: Chromosome 1 ‐ NC_000001.11 using assembly GRCh38.p14.

The *G6PC* gene encodes the enzyme of glucose 6‐phosphatase and mutations in the *G6PC* gene could cause an inherited disorder, glycogen storage disease type I (GSDI). In the present study, we found 38 PA variants in the *G6PC* gene exhibited CRP‐increasing effects (Figure S9; Table S8). The variant with the smallest *p* value (6.50e‐03) was a frameshift variant, 17:42900952:TC:T, with a total of 77 heterozygous carriers showing an increased serum CRP by 0.326 on log scale (equivalent to a 38.5% increase on mg/L scale). However, none of the PA variants in the *G6PC* gene reached the FDR significance level (Table S8).

Moreover, the sensitivity variant analysis of “leave‐one‐out” showed that none of the single variants could alter the effect of aggregated PA mutation in the *CRP* gene or the *G6PC* gene appreciably (Tables S9 and S10).

### 3.4 Interaction effect between PA mutation burden and obesity categories

Of the 161,430 participants included in the present study, 313 individuals (128 men and 185 women) are heterozygous carriers of any PA allele in the *CRP* gene. We compared the population characteristics at baseline by the status of PA mutation burden in the *CRP* gene, and found carriers and non‐carriers were not significantly different, except for the serum CRP concentration (Table S11). However, 663 carriers of PA alleles in the *G6PC* gene were different from the non‐carriers with respect to several baseline characteristics, including higher age, greater levels of systolic blood pressure, triglycerides, and serum CRP, as well as lower levels of BMI, waist circumference, and glucose (Table S12).

Using BMI and waist circumference, we observed that 23.5% and 32.9% of the study participants were classified as obese and had high central fat accumulation, respectively. The serum CRP concentrations at baseline were significantly different across strata by the PA mutation burden in the *CRP* and *G6PC* genes, BMI category, and waist circumference category (Tables S13 and S14). Modelling the effects of these factors individually on serum CRP, we found the obese status exhibited the largest effect with a 1.12 (1.10, 1.13) increase in natural logarithm of CRP concentration, followed by “high central fat accumulation” (0.94 [0.93, 0.95]), PA mutation burden in the *CRP* gene (−0.68 [−0.79, −0.56]), overweight (0.52 [0.50, 0.53]), underweight (−0.49 [−0.56, −0.42]), “moderate central fat accumulation” (0.44 [0.43, 0.45]) and PA mutation burden in the *G6PC* gene (0.17 [0.09, 0.25]; Table S11).

**FIGURE 3:**
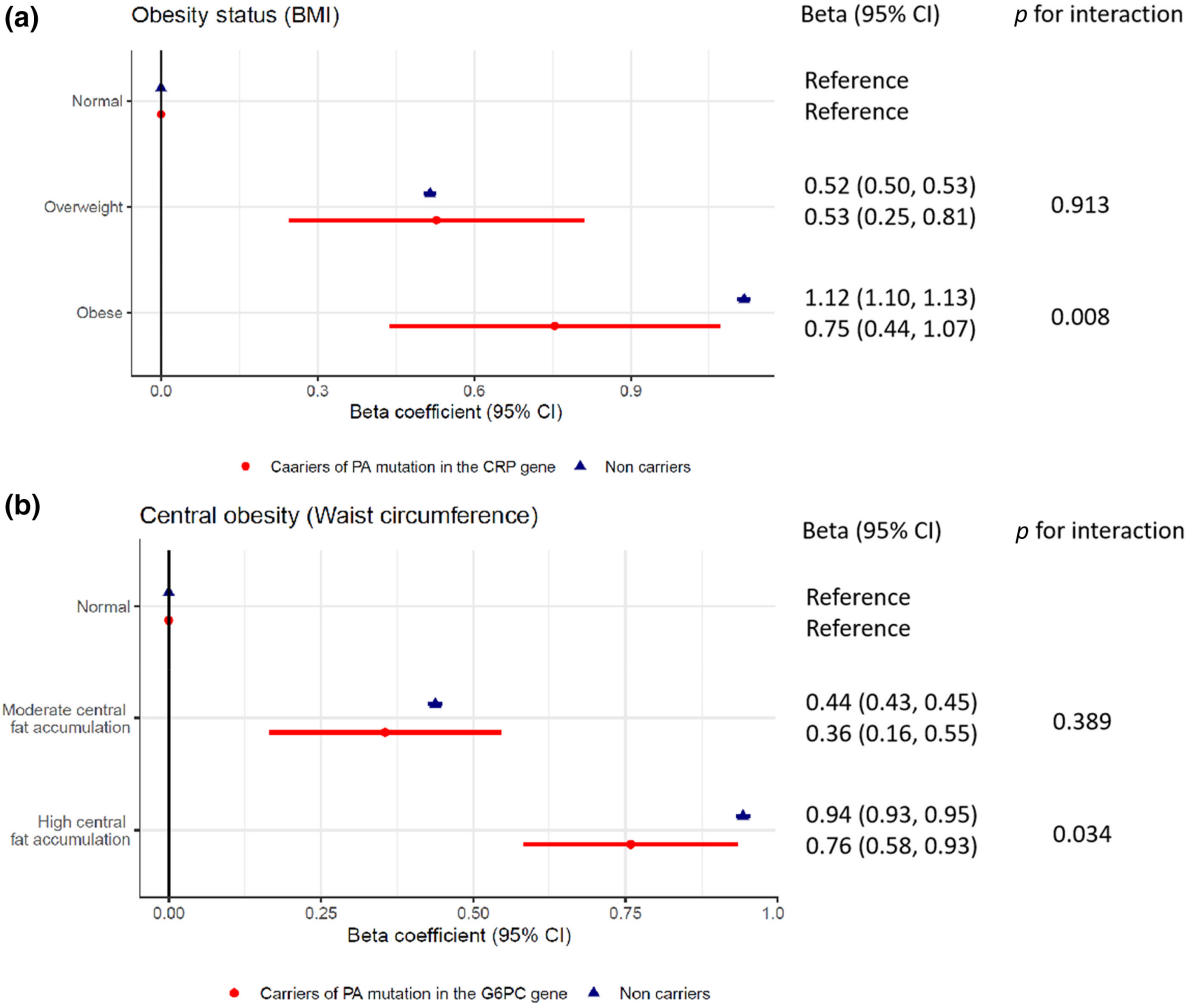
Associations between obesity, or central obesity, and serum CRP (natural log transformed) by PA mutation status in the *CRP* and *G6PC* genes. (a) illustrates the beta coefficients of overweight and obese status among carriers and non‐carriers of PA alleles in the *CRP* gene. Underweight participants were excluded from the analysis because of small sample size (as we only observed one PA mutation carrier in the *CRP* gene being underweight). The *p* values for interaction were tested by introducing the multiplicative terms between PA mutation burden in the *CRP* gene and the BMI categories. All models were adjusted for age, sex, birth year category, WES release, and 20 genomic PCs. (b) illustrates the beta coefficients of central obesity status among carriers and non‐carriers of PA alleles in the *G6PC* gene. Annotations in (b) are the same as in (a). *CRP* gene: Chromosome 1 ‐ NC_000001.11 using assembly GRCh38.p14; *G6PC* gene: Chromosome 11 ‐ NC_000077.7 using assembly GRCh38.p14.

Particularly, we observed a significant interaction effect between PA mutation burden in the *CRP* gene and obesity measured by BMI, as well as PA mutation burden in the *G6PC* gene and central obesity indexed by waist circumference. The relative effects of obesity on serum CRP in the PA mutation carriers were different from that in the non‐carriers. Specifically, obese status increased the log‐transformed concentration of serum CRP by 0.75 (0.44, 1.07) in the PA mutation carriers in the *CRP* gene, significantly smaller than an increase of 1.12 (1.10, 1.13) in the non‐carriers of PA alleles in the *CRP* gene (*p* for interaction = 0.008). Meanwhile, the CRP‐raising effect of high central fat accumulation was significantly different by the presence of PA mutation in the *G6PC* gene. High central fat accumulation carriers had smaller effect on serum CRP levels (*β* [95% CI] = 0.76 [0.58, 0.93]) than non‐carriers with high central fat accumulation (*β* [95% CI] = 0.94 [0.93, 0.95], Figure 3 and Table S16).

### 3.5 PA mutation burden and CRP‐associated diseases

We estimated the relative risk of 16 CRP‐associated diseases related to the carriers of PA alleles in the *CRP* and *G6PC* genes. Among the PA mutation carriers, we documented different numbers of cases across all the conditions, ranging from 1 (0.3% for celiac disease) to 33 (10.6% for depressive disorder) for the *CRP* gene and 1 (0.2% for psoriatic arthritis) to 68 (10.3% for depressive disorder) for the *G6PC* gene. However, we did not observe robust evidence to support a relationship across the health outcomes (Tables S17 and S18).

## 4 DISCUSSION

We explored rare functional variants using WES data in association with serum CRP among 161,430 UKB participants and found PA mutations in the *CRP* and the *G6PC* gene to significantly influence serum CRP concentration, by a 49.6% reduction and a 22.5% increase, respectively. Seven PA variants located in the *CRP* gene were individually associated with lower serum CRP levels, and those variants cannot be detected through array genotyping and imputation methods. We found the effect of PA mutation burden to be independent of common genetic variants. Particularly, obesity and central obesity influenced the serum CRP levels to a lesser extent in the carriers of the PA alleles in the *CRP* gene and the *G6PC* gene compared to the non‐carriers. In addition, we did not observe altered risks of CRP‐associated diseases among PA mutation carriers.

Several studies have tested rare functional variants at the gene level in association with thousands of phenotypes using different release rounds of WES data in UKB previously. (Backman et al., 2021; Van Hout et al., 2020; Wang et al., 2021) Of those studies, the one with the largest sample size used data from 454,787 individuals and found two exome‐wide significant genes, the *CRP* and *G6PC* genes, to be associated with serum CRP. (Backman et al., 2021) Particularly, these gene‐level associations were successfully replicated in another independent sample. (Backman et al., 2021) Using a smaller sample size, the present analysis also identified these two serum CRP‐associated genes in the discovery and replication sets. In our study, the *G6PC* gene passed the exome‐wide significance threshold only when BMI was adjusted for in the burden test, suggesting the identified effect of the *G6PC* gene was independent of BMI and accounting for BMI in our model could potentially increase the statistical power in the detection of serum CRP‐associated genetic variants.

The *CRP* gene is the direct coding sequence of the C‐reactive protein. We inspected the individual PA variants along the *CRP* gene region and identified seven variants that were individually associated with serum CRP at an FDR significance level. Start loss mutations affect the initiation codon and demonstrated the largest effect on serum CRP concentration in the present analysis, with seven carriers showing a 71% reduced serum CRP concentration on average compared to the non‐carriers. It is worth noting that all PA mutation carriers are heterozygotes, which in part explains the detection of circulating CRP in the presence of start loss mutation. Missense mutations result in the replacement of one amino acid with another and the pathogenicity of missense variants was predicted by computational algorithms using sequence‐based and structure‐based predictive features. (Adzhubei et al., 2010; Ng & Henikoff, 2003) Deleterious missense mutations could alter the physiochemical properties of the amino acids, subsequently leading to incorrect folding and decreased stability of the proteins. (Stefl et al., 2013) Besides, the amino acid substitution occurring at the signal peptides can mislocalize the protein to the wrong cellular part. We noticed that the carriers of four deleterious missense mutations expressed decreased CRP in the blood. Of those variants, the missense variant of 1:159714463:A:G influenced the signal peptide of CRP, which consists of 18 amino acids (Hage & Szalai, 2007), and 69 heterozygous mutation carriers showed a 48% decreased serum CRP level. Protein mislocation could be a possible explanation, and future investigations are needed.

The *G6PC* gene encodes the enzyme of glucose 6‐phosphatase, a key enzyme to maintain glucose homeostasis. Mutations in the *G6PC* gene were found to cause the glycogen storage disease type I (GSD1), a rare inborn metabolic disorder. (Kishnani et al., 2014) At the variant level, the top signal, frameshift variant 17:42900952:TC:T, was associated with increased serum CRP in the present study and is interpreted as the GSD1‐related mutations in the ClinVar database. (Medicine, 2022) GSD1 patients express excessive glycogen and fat in the liver and kidneys. (Kishnani et al., 2014) Considering that adipose tissue also produces CRP, accumulated fat might be the reason leading to an increased serum CRP concentration in the mutation carriers. Interestingly, the associations between the *G6PC* gene and serum CRP associations were observed for rare mutations only, but not for common genetic variants as the *G6PC* gene has not been reported in the previous GWASs for serum CRP. Since GSD1 is a rare disease, the increased blood CRP level in rare PA mutation carriers of the *G6PC* gene could be a long‐term consequence resulting from the progression of the disease. Evidence to elucidate the molecular mechanisms is warranted.

Moreover, we found a gene–environment interaction between rare variants and obesity. The effect of obesity in elevating serum CRP was smaller in the rare mutation carriers than in the non‐carriers. A similar interaction was demonstrated for common genetic variants of CRP in previous studies. (Curocichin et al., 2011; Dehghan et al., 2011; Eiriksdottir et al., 2009; Teng et al., 2009) Besides liver, adipose tissue is another major contributor to the production of CRP. (Anty et al., 2006) In the present study, due to the PA alleles in the *CRP* gene, heterozygous carriers could exhibit impaired ability to produce functional CRP in both liver and adipose tissue, therefore explaining a weaker effect of obesity on serum CRP for PA mutation carriers. Considering both serum CRP and obesity status play an important role in the risk assessment of cardiovascular disease, both genetic and environmental factors need to be considered when interpreting the health risks indicated by serum CRP levels in clinical settings, especially among rare mutation carriers.

Examining the clinical relevance of rare coding mutations could reveal therapeutic insights for disease treatment. We looked through a series of CRP‐associated diseases and did not find robust evidence for altered risks among PA mutation carriers. Using polygenic risk scores of serum CRP and the design of Mendelian randomization, the genetic propensity to higher serum CRP was associated with a reduced risk of schizophrenia and prostate cancer, as well as an increased risk of chronic obstructive pulmonary disease. (Said et al., 2022) However, due to the rarity of the PA alleles, the present study was likely to be underpowered in the detection of disease risks. Studies with larger sample sizes will be needed.

The present analysis comes with a few strengths, including conducting several sensitivity and follow‐up analyses to interpret the clinical relevance of PA mutations. We acknowledge several limitations. First, UKB participants were invited to the study in their middle age. Therefore, mutations that are not compatible with middle‐aged survival cannot be studied in the present analysis. Second, the disease diagnoses were ascertained from self‐reported information, inpatient data, and death register. Diseases that are mainly captured by out‐patient data could be missed. Third, analyses were restricted to white British ethnicity only.

In conclusion, we found rare PA mutation burdens in the *CRP* and *G6PC* genes to be strongly associated with altered serum CRP concentrations. The CRP level among rare functional mutation carriers was less influenced by obese status. As serum CRP and obesity status are important predictors of cardiovascular risks in clinics, our observations underscore the need to take rare genetic factors into consideration to improve the delivery of precision medicine.

## AUTHOR CONTRIBUTIONS

All authors contributed to the study conception and design. Data analysis was performed by Xia Li, Alexander Ploner, Yunzhang Wang, and Jonathan K. L. Mak. The first draft of the manuscript was written by Xia Li and Sara Hägg and all authors commented on previous versions of the manuscript. All authors read and approved the final manuscript.

## FUNDING INFORMATION

This work was supported by the Swedish Research Council (2015‐03255, 2018‐02077), FORTE (2013‐2292), the Loo & Hans Osterman Foundation, the Foundation for Geriatric Diseases, the Magnus Bergwall Foundation, the Strategic Research Program in Epidemiology at Karolinska Institutet, King Gustaf V and Queen Victoria's Foundation of Freemasons, the China Scholarship Council, and the Swedish National Graduate School for Competitive Science on Aging and Health. The Swedish Twin Registry is managed by Karolinska Institutet and receives funding as an infrastructure through the Swedish Research Council, 2017‐00641. The analyses on UK Biobank genotypes were enabled by resources provided by the Swedish National Infrastructure for Computing (SNIC) at Uppmax partially funded by the Swedish Research Council through grant agreement no. 2018‐05973.

## CONFLICT OF INTEREST STATEMENT

The authors have no relevant financial or non‐financial interests to disclose.

## ETHICS STATEMENT

UK Biobank had obtained ethics approval from the North West Multicentre Research Ethics Committee (11/NW/0382 and 16/NW/0274) and informed consent from all participants, which covered the approved use of the data in the present analyses (Approved Research ID: 22224).

## CONSENT TO PARTICIPATE

Informed consent was obtained from the UKB participants included in the present study.

## Supporting information


Data S1.

Data S2.

## Data Availability

Pseudonymized individual‐level data in the present study are publicly available to registered researchers and can be accessed through an application to the UK Biobank. This research has been conducted using the UK Biobank Resource under Application Number ‘22224’. The summary statistics of the gene‐based burden test generated in this study are presented in the Data S2.
