# Supplementary material for: Rare functional variants in the CRP and G6PC genes modify the relationship between obesity and serum C‐reactive protein in white British population
Source: Mol Genet Genomic Med. 2023 Jul 26;11(12):e2255. doi: 10.1002/mgg3.2255 (PMC10724514; doi:10.1002/mgg3.2255)
Supplement: Supplementary file 1 — Data S1. [file MGG3-11-e2255-s001.docx]

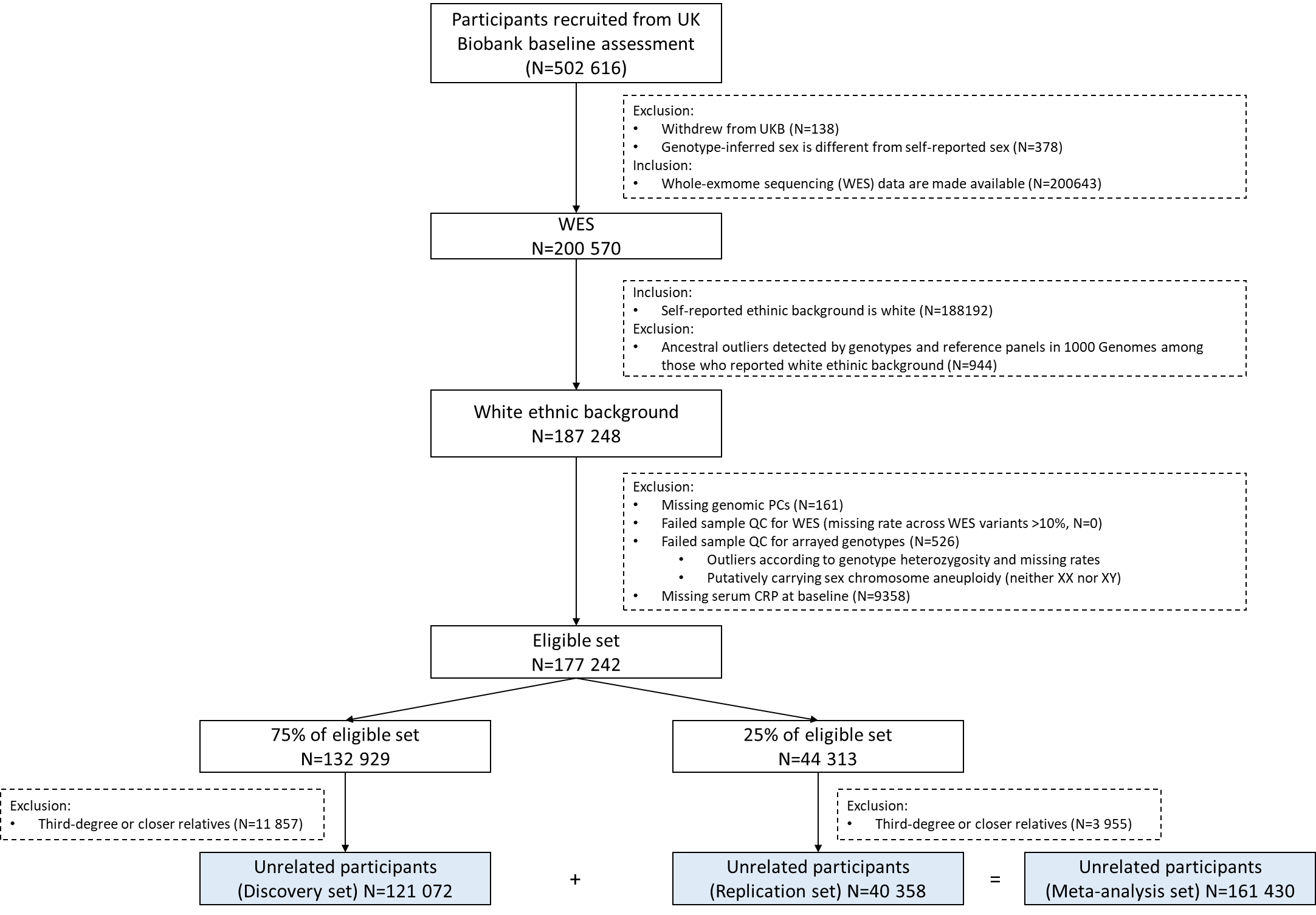


Fig.S1 Study population


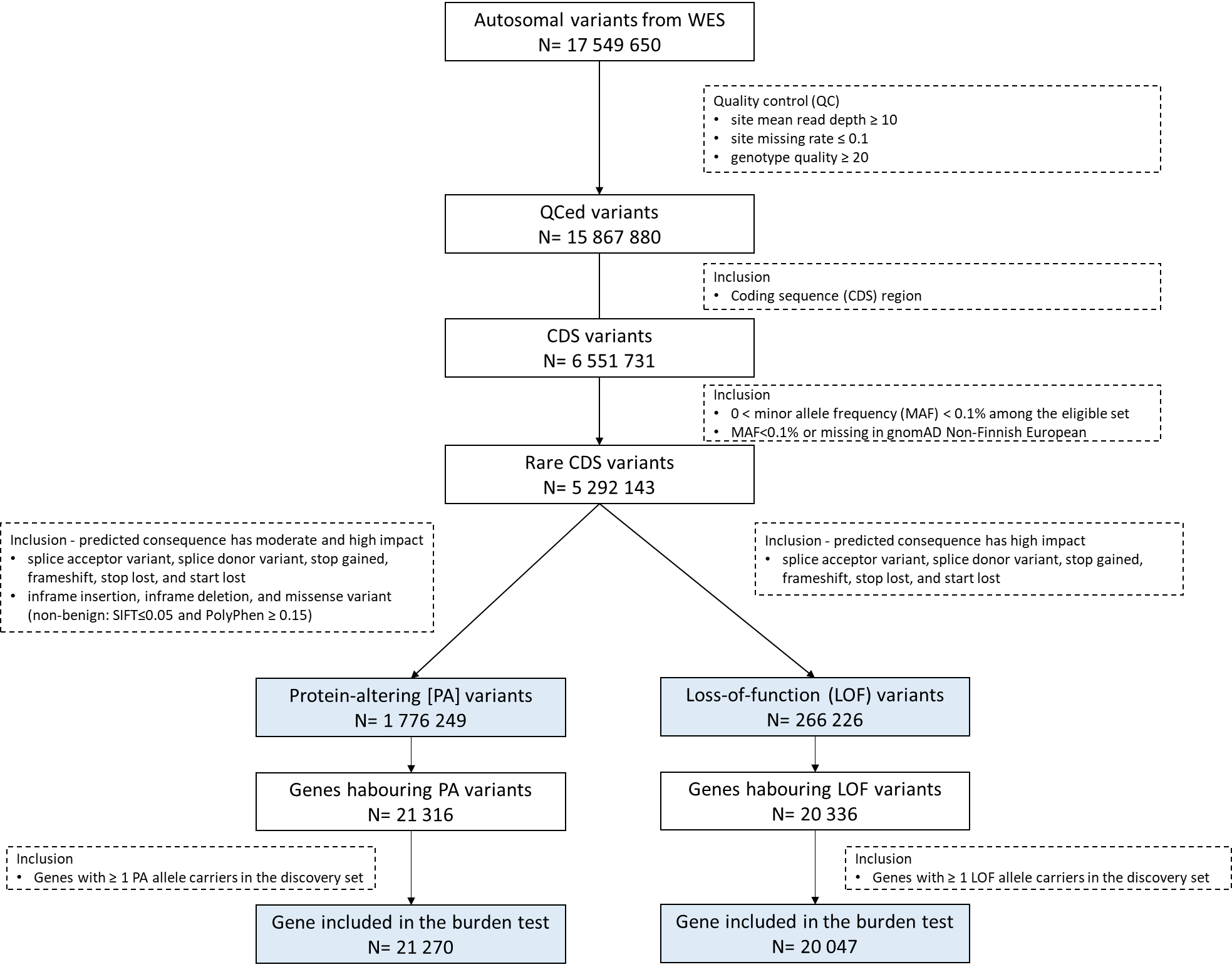


Fig.S2 Genetic variants and genes


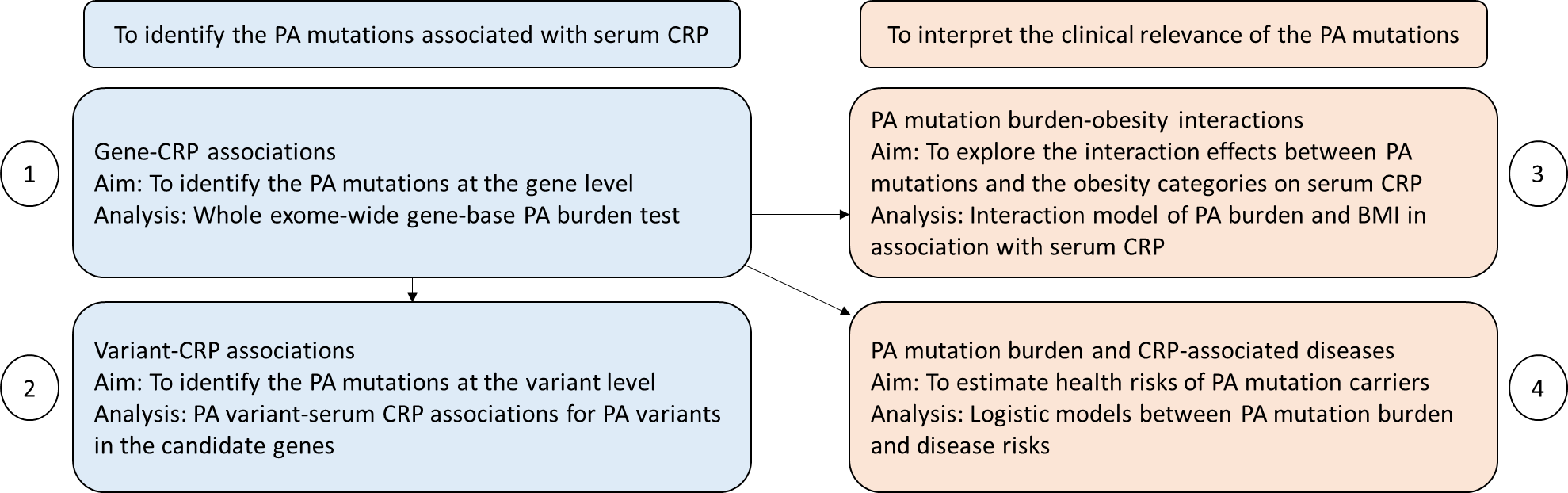


Fig.S3 Overview of the study aims and the analysis plan

Table S1 Disease ascertainment

|  | Values in Field 20002 | ICD9 codes | ICD10 codes |
| --- | --- | --- | --- |
| **Autoimmune/inflammatory** | |  |  |
| Celiac disease |  | 579.0 | K90.0 |
| IBD (all types) | 1461-1463 | 555-558 | K50-52 |
| Crohn’s disease | 1462 | 555 | K50 |
| Ulcerative colitis | 1463 | 556 | K51 |
| Psoriatic arthritis |  | 696.0 | L40.52 |
| Rheumatoid arthritis | 1464 | 7140, 7141, 7142, 7149 | M05, M06 |
| Type 1 diabetes | 1222 | 250.01,250.03,250.11,250.13,250.21,250.23,250.31,250.33,250.41,250.43,250.51,250.53,250.61,250.63,250.71,250.73,250.81,250.83,250.91,250.93 | E10 |
| Knee osteoarthritis |  | 715.96 | M17 |
| **Cardiovascular** |  |  |  |
| Coronary artery disease | 1075 | 414 | I25 |
| Ischemic stroke (all type) | 1583 | 434 | I63 |
| **Metabolic** |  |  |  |
| Type 2 diabetes | 1223 | 250.00,250.02,250.10,250.12,250.20,250.22,250.30,250.32,250.40,250.42,250.50,250.52,250.60,250.62,250.70,250.72,250.80,250.82,250.90,250.92 | E11 |
| Chronic kidney disease | 1192-1194 | 585 | N18 |
| **Neurodegenerative** |  |  |  |
| Alzheimer disease | 1263 | 331.0 | G30 |
| Parkinson disease | 1262 | 332 | G20 |
| **Psychiatric** |  |  |  |
| Bipolar disorder | 1291 | 269.0-296.6 | F31 |
| Depressive disorder | 1286 | 296.2-296.3 | F32-F33 |

Table S2 Characteristics of PA and LOF variants analyzed

|  | PA variants | |  | LOF variants | |
| --- | --- | --- | --- | --- | --- |
|  | Number | Proportion |  | Number | Proportion |
| Number of variants | 1,776,249 |  |  | 266,226 |  |
| Consequence type | |  |  |  |  |
| Missense variant | 1,451,473 | 81.7% |  |  |  |
| Frameshift variant | 143,584 | 8.1% |  | 143,584 | 53.9% |
| Stop gained | 112,504 | 6.3% |  | 112,504 | 42.3% |
| Inframe deletion | 40,124 | 2.3% |  |  |  |
| Inframe insertion | 18,013 | 1.0% |  |  |  |
| Start lost | 4,875 | 0.3% |  | 4,875 | 1.8% |
| Stop lost | 3,049 | 0.2% |  | 3,049 | 1.1% |
| Splice donor variant | 1,407 | 0.1% |  | 1,407 | 0.5% |
| Splice acceptor variant and others | 1,220 | <0.1% |  | 807 | 0.3% |
| MAF category | |  |  |  |  |
| [0, 0.0001%] | 10 | <0.1% |  | 6 | <0.1% |
| (0.0001%, 0.001%] | 1,247,252 | 70.2% |  | 201,934 | 75.9% |
| (0.001%, 0.01%] | 473,546 | 26.7% |  | 58,359 | 21.9% |
| (0.01%, 0.1%] | 55,441 | 3.1% |  | 5,927 | 2.2% |

PA protein-altering, LOF loss of function, MAF minor allele frequency.

Table S3 Characteristics of genes analyzed in the burden tests

|  | PA-harboring genes | LOF-harboring genes |
| --- | --- | --- |
| Number of mapped genes | 21316 | 20336 |
| Number of genes analyzed in the present analysis | 21270 | 20047 |
| Gene length in bp, median(IQR) | 27278 (61262) | 28523 (62908) |
| Category of gene length in bp, N(proportion) | | |
| (10,100] | 38(0.2%) | 17(0.1%) |
| (100,1e+03] | 517(2.4%) | 419(2.1%) |
| (1e+03,1e+04] | 4900(23.0%) | 4427(22.1%) |
| (1e+04,1e+05] | 11975(56.3%) | 11453(57.1%) |
| (1e+05,1e+06] | 3773(17.7%) | 3666(18.3%) |
| (1e+06,1e+07] | 67(0.3%) | 65(0.3%) |
| Number of PA/LOF variants per gene, median(IQR) | 71 (87) | 12 (14) |
| Category of PA/LOF variants per gene, N(proportion) | | |
| (0,10] | 1415(6.7%) | 9174(45.8%) |
| (10,100] | 12636(59.4%) | 10742(53.6%) |
| (100,1e+03] | 7160(33.7%) | 131(0.7%) |
| (1e+03,1e+04] | 59(0.3%) |  |
| Number of mutation carriers per gene in the discover set, median(IQR) | 282 (422) | 25 (55) |
| Category of mutation carrier per gene in the discover set, N(proportion) | | |
| (0,10] | 707(3.3%) | 5867(29.3%) |
| (10,100] | 3899(18.3%) | 11035(55.0%) |
| (100,1e+03] | 14936(70.2%) | 3120(15.6%) |
| (1e+03,1e+04] | 1709(8.0%) | 25(0.1%) |
| (1e+04,1e+05] | 19(0.1%) |  |

PA protein-altering, LOF loss of function, MAF minor allele frequency, bp base-pair, IQR interquartile range.


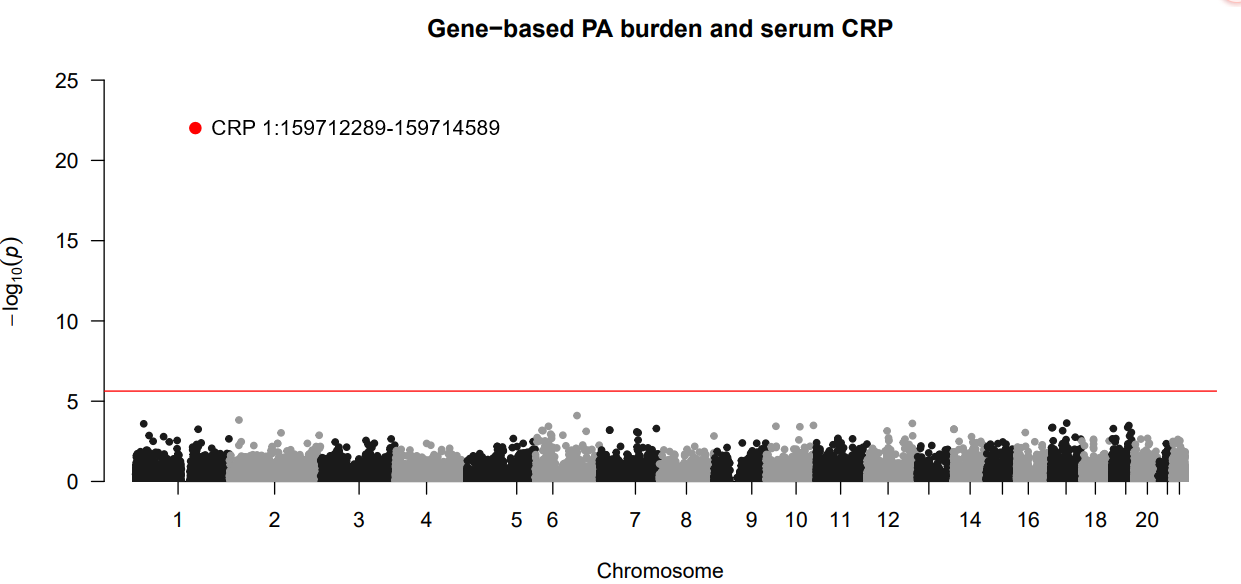


Fig.S4 Associations between gene-based PA mutation burden and serum CRP in the discovery set (Model 1 of CMC burden tests)

The Manhattan plot shows the associations between gene-based PA mutation burden and serum CRP (natural-log transformed), estimated by CMC burden test in the discovery set. Age, sex, WES release information, and 20 genomic PCs were controlled in the statistical models, i.e., Model 1. Each dot represents a gene, of which the location is determined by the chromosome and position on X scale and the statistic value of –log10(P) on Y scale. The red horizontal line denotes the whole exome-wide significant level (P< 2.35E-06). Annotations, including name, chromosome, and location, of the exome-wide significant genes were displayed in the plot.

Table S4 Whole exome-wide significant associations between PA/LOF mutation burden and natural log-transformed serum CRP concentration (Model 1 of CMC burden tests)

| Mutation and Population | Number of individuals | Number of PA/LOF variants in the *CRP/G6PC* gene | Number of carriers of PA/LOF alleles in the *CRP/G6PC* gene | Beta (SE) | P value |
| --- | --- | --- | --- | --- | --- |
| PA mutation in the *CRP* gene | | | | | |
| Discovery | 121072 | 52 | 230 | -0.676(0.069) | 9.75e-23 |
| Replication | 40358 | 52 | 83 | -0.676(0.115) | 4.15e-09 |
| PA mutation in the *G6PC* gene | | | | | |
| Discovery | 121072 | 76 | 500 | 0.172(0.047) | 2.32e-04 |
| Replication | 40358 | 76 | 163 | 0.172(0.082) | 3.66e-02 |
| LOF mutation in the *CRP* gene | | | | | |
| Discovery | 121072 | 12 | 64 | -0.724(0.131) | 2.89e-08 |
| Replication | 40358 | 12 | 25 | -0.721(0.209) | 5.74e-04 |

Beta regression coefficient of the PA mutation burden, SE standard error. Age, sex, WES release information, and 20 genomic PCs were controlled in the statistical models, i.e., Model 1.


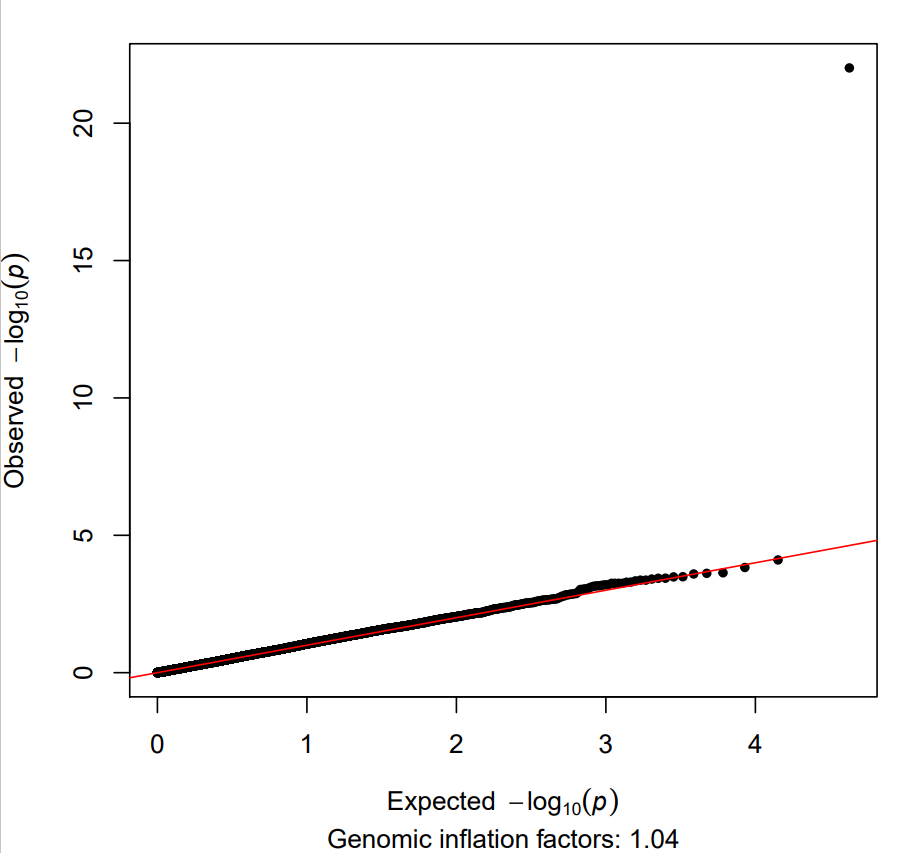


Fig.S5 Quantile-quantile (Q–Q) plot showing the observed statistics (PA mutation burden tests in the discovery set) against the expected statistics under the null hypothesis of no association

Age, sex, WES release information, and 20 genomic PCs were controlled in the statistical models.

Table S5 Associations between PA mutation burden in the CRP gene and serum CRP (natural-log transformed) in the meta-analysis set with adjustment for the PRS of CRP

| PA mutation burden | Number of mutation carriers | Effect size (Beta) | Standard error | P value |
| --- | --- | --- | --- | --- |
| In the *CRP* gene | 312 | -0.679 | 0.052 | 1.29e-39 |
| In the *G6PC* gene | 659 | 0.234 | 0.036 | 4.57e-11 |

Age, sex, WES release information, 20 genomic PCs, and BMI were controlled in the statistical models.


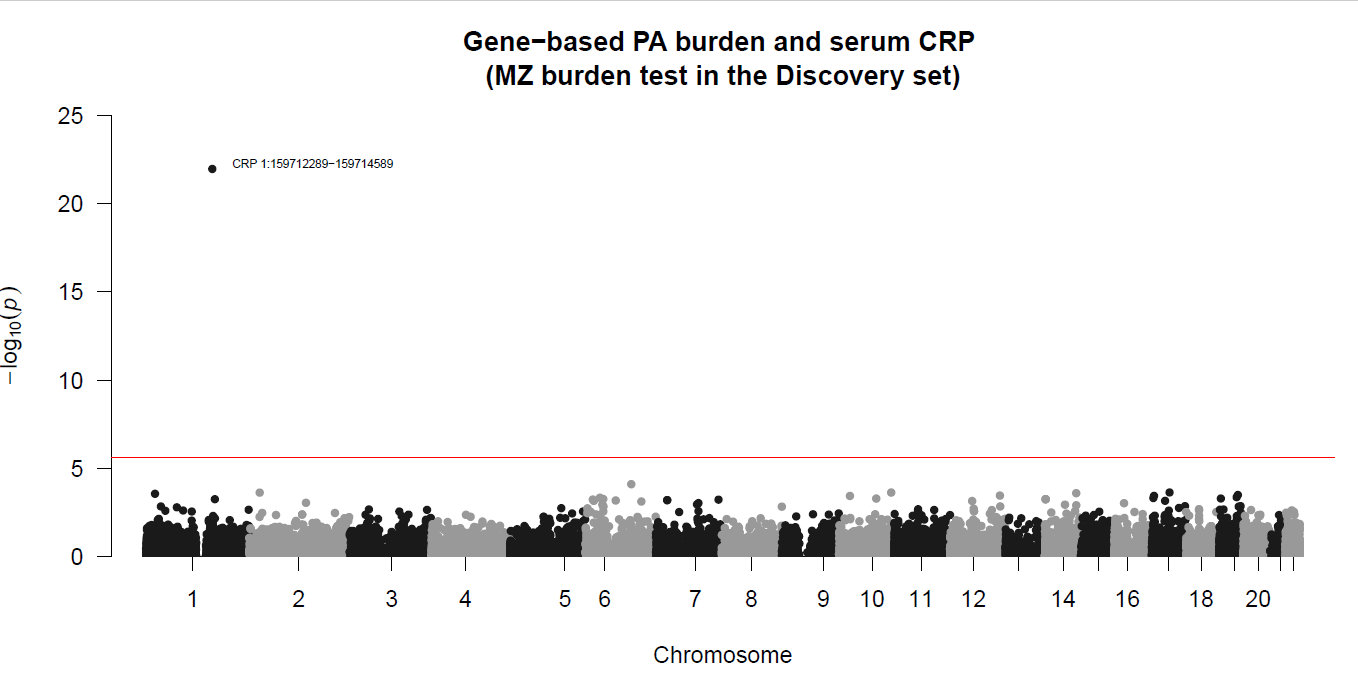


Fig.S6 Associations between gene-based PA mutation burden and serum CRP (natural-log transformed) in the discovery set (MZ burden test)

The Manhattan plot shows the associations between gene-based PA mutation burden and serum CRP (natural-log transformed), estimated by MZ burden test in the discovery set. Age, sex, WES release information, and 20 genomic PCs were controlled in the statistical models. Each dot represents a gene, of which the location is determined by the chromosome and position on X scale and the statistic value of –log10(P) on Y scale. The red horizontal line denotes the whole exome-wide significant level.


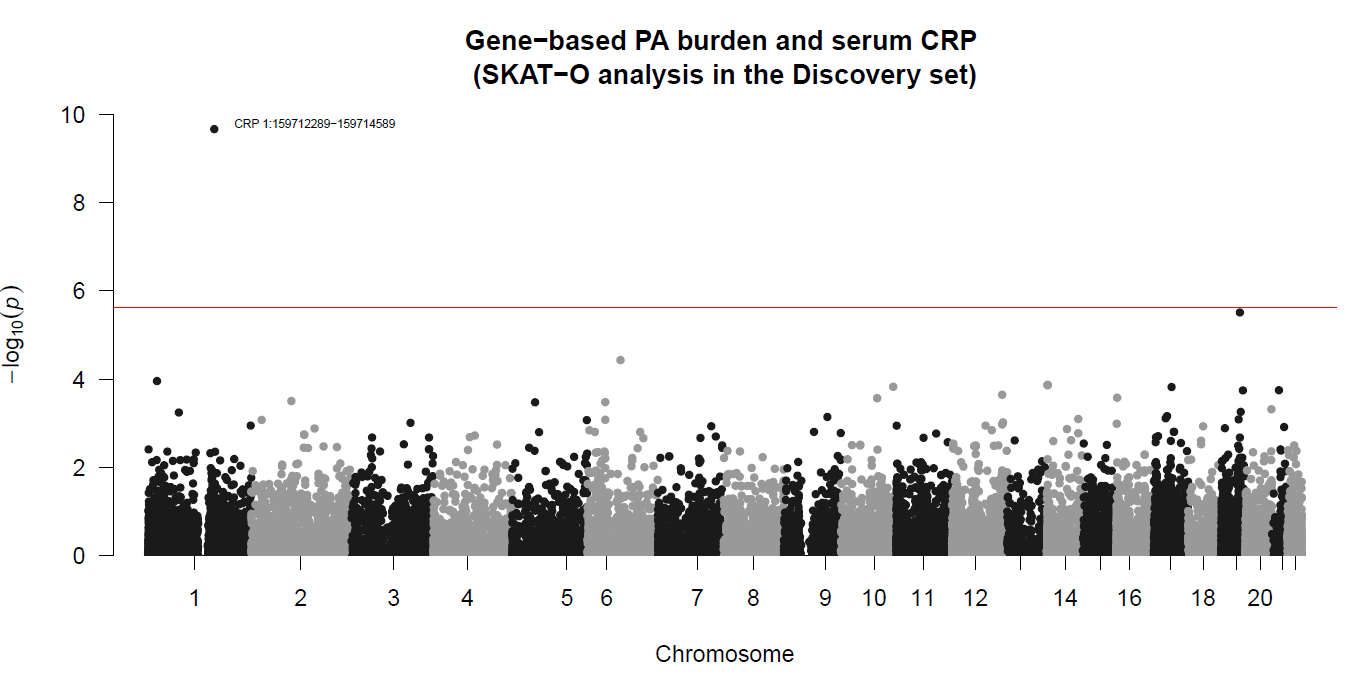


Fig.S7 Associations between gene-based PA mutation burden and serum CRP (natural-log transformed) in the discovery set (SKAT-O test)

The Manhattan plot shows the associations between gene-based PA mutation burden and serum CRP (natural-log transformed), estimated by SKAT-O test in the discovery set. Age, sex, WES release information, and 20 genomic PCs were controlled in the statistical models. Each dot represents a gene, of which the location is determined by the chromosome and position on X scale and the statistic value of –log10(P) on Y scale. The red horizontal line denotes the whole exome-wide significant level.


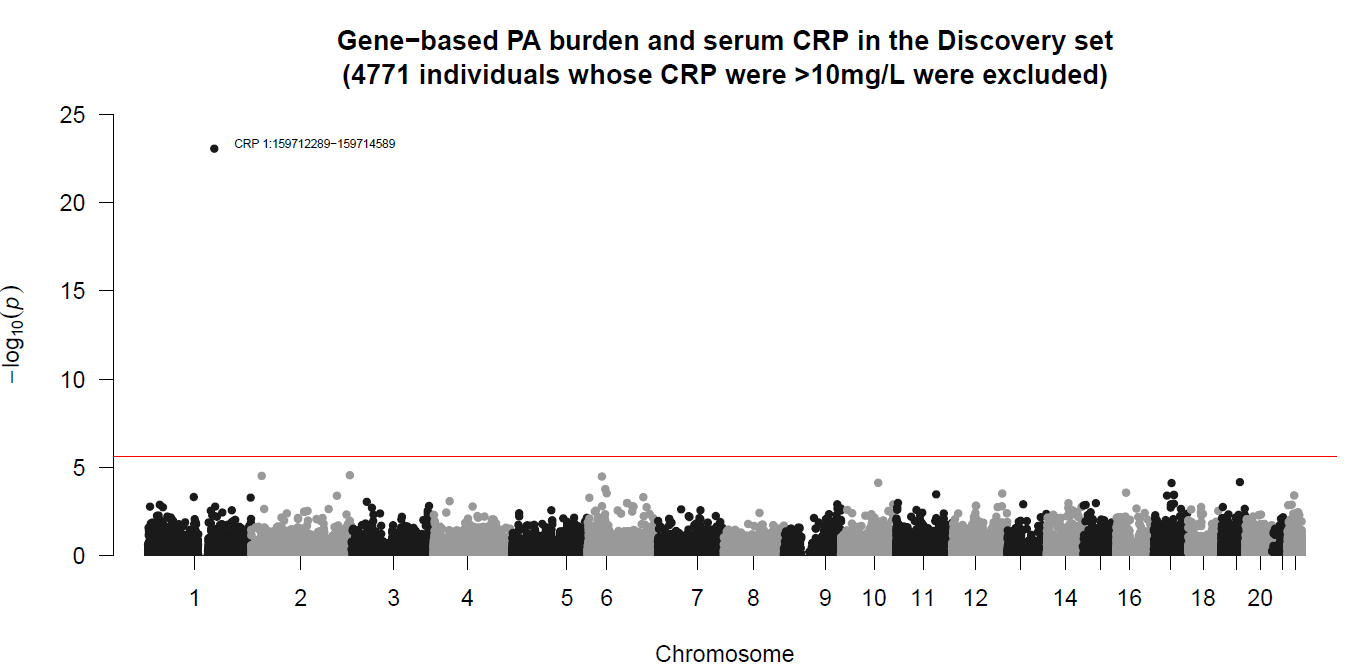


Fig.S8 Associations between gene-based PA mutation burden and serum CRP (natural-log transformed) in the discovery set with individuals whose baseline CRP concentration were higher than 10mg/L excluded (CMC burden test)

The Manhattan plot shows the associations between gene-based PA mutation burden and serum CRP (natural-log transformed), estimated by CMC burden test in the discovery set and individuals whose baseline CRP concentration were higher than 10mg/L were excluded. Age, sex, WES release information, and 20 genomic PCs were controlled in the statistical models. Each dot represents a gene, of which the location is determined by the chromosome and position on X scale and the statistic value of –log10(P) on Y scale. The red horizontal line denotes the whole exome-wide significant level.

Table S6 Associations between PA variants in the coding sequence of *CRP* gene and serum CRP (natural-log transformed) in the meta-analysis set

| variant_id | reference_allele | alternate_allele | transcript | Consequense | CDS_position | Amino_acids | number_of_ref_allele_homozygote | number_of_heterozygote | number_of_alt_allele_homozygote | alt_allele_frequency | alt_allele_beta(se) | p_value | FDR p value |
| --- | --- | --- | --- | --- | --- | --- | --- | --- | --- | --- | --- | --- | --- |
| 1:159714463:A:G | A | G | ENST00000255030 | Missense variant | 23 | L/S | 161361 | 69 | 0 | 2.14e-04 | -0.657(0.126) | 1.76e-07 | 8.64e-06 |
| 1:159714026:G:C | G | C | ENST00000255030 | Stop gained | 174 | Y/* | 161366 | 64 | 0 | 1.98e-04 | -0.65(0.131) | 6.40e-07 | 1.57e-05 |
| 1:159714007:G:A | G | A | ENST00000255030 | Missense variant | 193 | R/C | 161412 | 18 | 0 | 5.58e-05 | -1.074(0.246) | 1.30e-05 | 2.13e-04 |
| 1:159713719:C:T | C | T | ENST00000255030 | Missense variant | 481 | G/S | 161401 | 29 | 0 | 8.98e-05 | -0.672(0.194) | 5.33e-04 | 6.53e-03 |
| 1:159714484:A:G | A | G | ENST00000255030 | Start lost | 2 | M/T | 161423 | 7 | 0 | 2.17e-05 | -1.251(0.395) | 1.54e-03 | 1.33e-02 |
| 1:159713583:C:G | C | G | ENST00000255030 | Missense variant | 617 | R/P | 161420 | 10 | 0 | 3.10e-05 | -1.041(0.33) | 1.63e-03 | 1.33e-02 |
| 1:159713531:C:T | C | T | ENST00000255030 | Stop gained | 669 | W/* | 161422 | 8 | 0 | 2.48e-05 | -1.07(0.369) | 3.77e-03 | 2.64e-02 |
| 1:159713869:G:C | G | C | ENST00000255030 | Missense variant | 331 | P/A | 161428 | 2 | 0 | 6.19e-06 | -1.703(0.739) | 2.12e-02 | 1.30e-01 |
| 1:159713988:G:A | G | A | ENST00000255030 | Missense variant | 212 | S/L | 161423 | 7 | 0 | 2.17e-05 | -0.869(0.395) | 2.77e-02 | 1.49e-01 |
| 1:159713896:A:G | A | G | ENST00000255030 | Missense variant | 304 | F/L | 161417 | 13 | 0 | 4.03e-05 | -0.62(0.29) | 3.24e-02 | 1.49e-01 |
| 1:159713733:T:C | T | C | ENST00000255030 | Missense variant | 467 | E/G | 161429 | 1 | 0 | 3.10e-06 | -2.221(1.045) | 3.35e-02 | 1.49e-01 |
| 1:159714066:G:A | G | A | ENST00000255030 | Missense variant | 134 | T/M | 161402 | 28 | 0 | 8.67e-05 | -0.412(0.197) | 3.71e-02 | 1.52e-01 |
| 1:159713822:C:A | C | A | ENST00000255030 | Missense variant | 378 | E/D | 161428 | 2 | 0 | 6.19e-06 | -1.457(0.739) | 4.85e-02 | 1.83e-01 |
| 1:159713663:C:A | C | A | ENST00000255030 | Missense variant | 537 | M/I | 161429 | 1 | 0 | 3.10e-06 | -1.992(1.045) | 5.66e-02 | 1.98e-01 |
| 1:159713664:A:G | A | G | ENST00000255030 | Missense variant | 536 | M/T | 161427 | 3 | 0 | 9.29e-06 | -1.084(0.603) | 7.24e-02 | 2.37e-01 |
| 1:159714136:T:C | T | C | ENST00000255030 | Missense variant | 64 | M/V | 161429 | 1 | 0 | 3.10e-06 | -1.809(1.045) | 8.34e-02 | 2.55e-01 |
| 1:159714473:A:C | A | C | ENST00000255030 | Missense variant | 13 | L/V | 161428 | 2 | 0 | 6.19e-06 | -0.981(0.739) | 1.84e-01 | 5.32e-01 |
| 1:159713972:I:1 | T | TG | ENST00000255030 | Frameshift variant | 227-228 | R/SX | 161429 | 1 | 0 | 3.10e-06 | -1.331(1.045) | 2.03e-01 | 5.43e-01 |
| 1:159713778:D:9 | TTCTTCAGAC | T | ENST00000255030 | Inframe deletion | 413-421 | SLKK/K | 161429 | 1 | 0 | 3.10e-06 | -1.29(1.045) | 2.17e-01 | 5.43e-01 |
| 1:159714112:G:C | G | C | ENST00000255030 | Missense variant | 88 | P/A | 161429 | 1 | 0 | 3.10e-06 | -1.277(1.045) | 2.22e-01 | 5.43e-01 |
| 1:159713764:D:2 | CCA | C | ENST00000255030 | Frameshift variant | 434-435 | V/X | 161429 | 1 | 0 | 3.10e-06 | -1.21(1.045) | 2.47e-01 | 5.76e-01 |
| 1:159713624:G:C | G | C | ENST00000255030 | Missense variant | 576 | I/M | 161427 | 3 | 0 | 9.29e-06 | 0.671(0.603) | 2.66e-01 | 5.92e-01 |
| 1:159714102:G:A | G | A | ENST00000255030 | Missense variant | 98 | S/L | 161429 | 1 | 0 | 3.10e-06 | -1.112(1.045) | 2.87e-01 | 6.04e-01 |
| 1:159713679:A:G | A | G | ENST00000255030 | Missense variant | 521 | I/T | 161429 | 1 | 0 | 3.10e-06 | -1.092(1.045) | 2.96e-01 | 6.04e-01 |
| 1:159713660:C:T | C | T | ENST00000255030 | Stop gained | 540 | W/* | 161429 | 1 | 0 | 3.10e-06 | -1.04(1.045) | 3.19e-01 | 6.26e-01 |
| 1:159713950:A:T | A | T | ENST00000255030 | Missense variant | 250 | F/I | 161429 | 1 | 0 | 3.10e-06 | -0.977(1.045) | 3.50e-01 | 6.45e-01 |
| 1:159713975:C:A | C | A | ENST00000255030 | Missense variant | 225 | K/N | 161427 | 3 | 0 | 9.29e-06 | -0.558(0.603) | 3.55e-01 | 6.45e-01 |
| 1:159713584:G:A | G | A | ENST00000255030 | Missense variant | 616 | R/W | 161429 | 1 | 0 | 3.10e-06 | -0.741(1.045) | 4.78e-01 | 7.76e-01 |
| 1:159714124:C:A | C | A | ENST00000255030 | Missense variant | 76 | A/S | 161428 | 2 | 0 | 6.19e-06 | -0.516(0.739) | 4.85e-01 | 7.76e-01 |
| 1:159713863:G:A | G | A | ENST00000255030 | Missense variant | 337 | H/Y | 161428 | 2 | 0 | 6.19e-06 | -0.509(0.739) | 4.91e-01 | 7.76e-01 |
| 1:159713827:C:T | C | T | ENST00000255030 | Missense variant | 373 | V/M | 161428 | 2 | 0 | 6.19e-06 | -0.509(0.739) | 4.91e-01 | 7.76e-01 |
| 1:159714006:C:T | C | T | ENST00000255030 | Missense variant | 194 | R/H | 161426 | 4 | 0 | 1.24e-05 | -0.34(0.522) | 5.15e-01 | 7.89e-01 |
| 1:159714025:T:C | T | C | ENST00000255030 | Missense variant | 175 | T/A | 161424 | 6 | 0 | 1.86e-05 | 0.264(0.427) | 5.35e-01 | 7.93e-01 |
| 1:159714105:T:C | T | C | ENST00000255030 | Missense variant | 95 | E/G | 161428 | 2 | 0 | 6.19e-06 | -0.399(0.739) | 5.89e-01 | 7.93e-01 |
| 1:159713991:A:G | A | G | ENST00000255030 | Missense variant | 209 | F/S | 161428 | 2 | 0 | 6.19e-06 | -0.399(0.739) | 5.89e-01 | 7.93e-01 |
| 1:159713663:C:T | C | T | ENST00000255030 | Missense variant | 537 | M/I | 161429 | 1 | 0 | 3.10e-06 | -0.561(1.045) | 5.91e-01 | 7.93e-01 |
| 1:159713525:T:A | T | A | ENST00000255030 | Stop lost | 675 | */C | 161429 | 1 | 0 | 3.10e-06 | -0.55(1.045) | 5.98e-01 | 7.93e-01 |
| 1:159713874:I:1 | A | AG | ENST00000255030 | Frameshift variant | 325-326 | V/AX | 161429 | 1 | 0 | 3.10e-06 | -0.386(1.045) | 7.12e-01 | 8.99e-01 |
| 1:159713912:D:1 | AC | A | ENST00000255030 | Frameshift variant | 287 | G/X | 161429 | 1 | 0 | 3.10e-06 | -0.381(1.045) | 7.15e-01 | 8.99e-01 |
| 1:159713952:A:G | A | G | ENST00000255030 | Missense variant | 248 | I/T | 161429 | 1 | 0 | 3.10e-06 | -0.21(1.045) | 8.41e-01 | 9.80e-01 |
| 1:159713728:C:A | C | A | ENST00000255030 | Missense variant | 472 | D/Y | 161429 | 1 | 0 | 3.10e-06 | 0.206(1.045) | 8.44e-01 | 9.80e-01 |
| 1:159713803:I:4 | G | GCTTC | ENST00000255030 | Frameshift variant | 396-397 | -/EX | 161428 | 2 | 0 | 6.19e-06 | -0.112(0.739) | 8.79e-01 | 9.80e-01 |
| 1:159714089:A:T | A | T | ENST00000255030 | Stop gained | 111 | Y/* | 161429 | 1 | 0 | 3.10e-06 | -0.146(1.045) | 8.89e-01 | 9.80e-01 |
| 1:159714081:A:G | A | G | ENST00000255030 | Missense variant | 119 | L/P | 161429 | 1 | 0 | 3.10e-06 | 0.122(1.045) | 9.07e-01 | 9.80e-01 |
| 1:159713560:C:T | C | T | ENST00000255030 | Missense variant | 640 | G/S | 161429 | 1 | 0 | 3.10e-06 | 0.081(1.045) | 9.38e-01 | 9.80e-01 |
| 1:159713790:T:G | T | G | ENST00000255030 | Missense variant | 410 | K/T | 161429 | 1 | 0 | 3.10e-06 | -0.073(1.045) | 9.44e-01 | 9.80e-01 |
| 1:159713791:T:A | T | A | ENST00000255030 | Stop gained | 409 | K/* | 161429 | 1 | 0 | 3.10e-06 | -0.073(1.045) | 9.44e-01 | 9.80e-01 |
| 1:159713739:C:T | C | T | ENST00000255030 | Missense variant | 461 | G/E | 161429 | 1 | 0 | 3.10e-06 | 0.052(1.045) | 9.60e-01 | 9.80e-01 |
| 1:159713673:T:A | T | A | ENST00000255030 | Missense variant | 527 | N/I | 161429 | 1 | 0 | 3.10e-06 | -0.012(1.045) | 9.91e-01 | 9.91e-01 |
| 1:159713542:G:A | G | A | ENST00000255030 | Missense variant | 658 | P/S | 161430 | 0 | 0 | 0.00e+00 |  |  |  |
| 1:159713830:T:C | T | C | ENST00000255030 | Missense variant | 370 | I/V | 161430 | 0 | 0 | 0.00e+00 |  |  |  |
| 1:159713967:T:C | T | C | ENST00000255030 | Missense variant | 233 | D/G | 161430 | 0 | 0 | 0.00e+00 |  |  |  |

Table S7 Associations between rs1205 and serum CRP (natural-log transformed) in the meta-analysis set

| Common variant | Number of TT/TC/CC | Effect allele | Per-allele effect size | Standard error | P value |
| --- | --- | --- | --- | --- | --- |
| Rs1205 C>T | 17644/71370/72416 | T | -0.175 | 0.004 | <1E-16 |


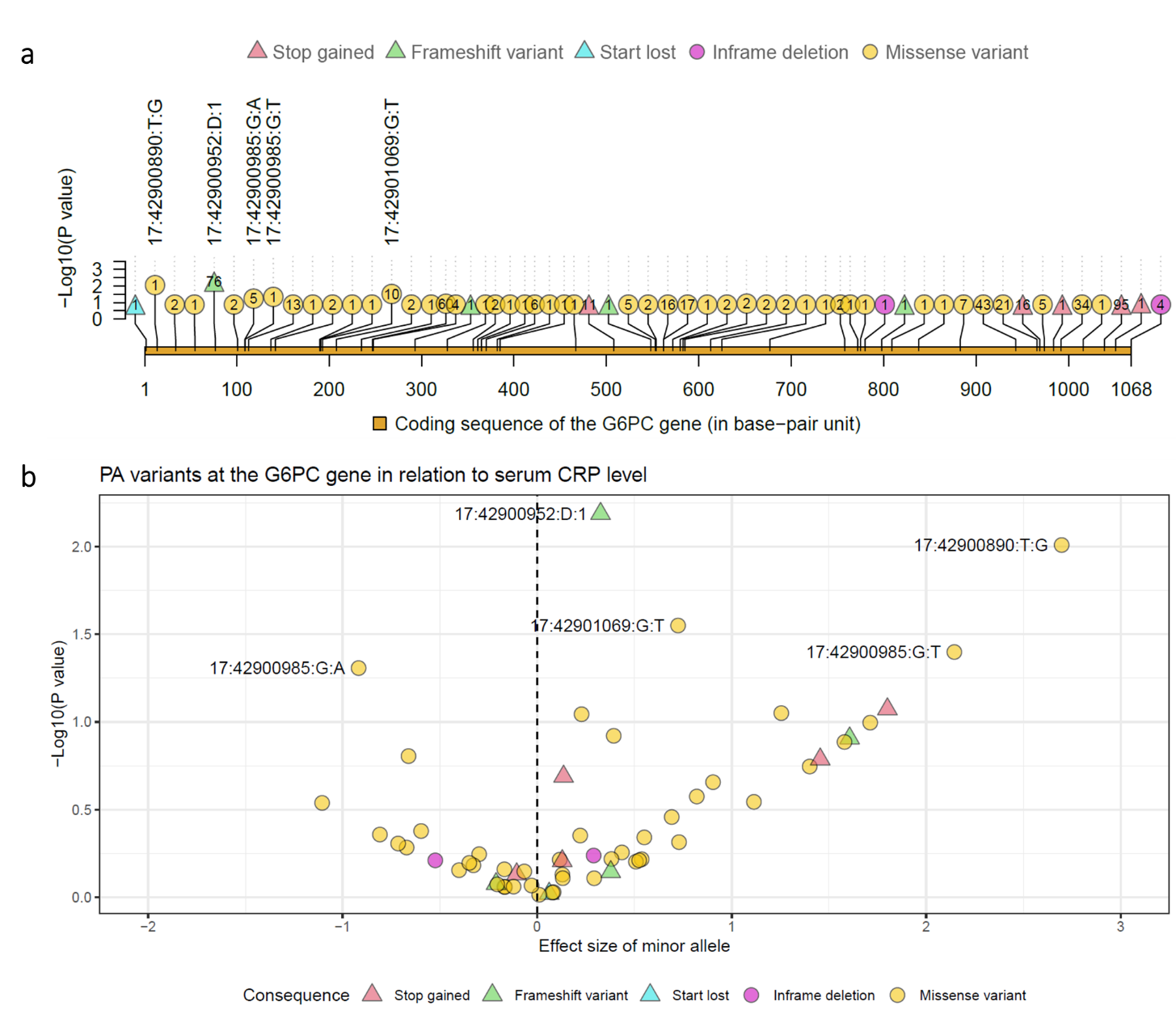


Fig.S9 PA variants in the coding sequence of the *G6PC* gene and the associations with serum CRP in the meta-analysis set

A The plot shows the location of PA variants in the coding sequence region of the *G6PC* gene on X scale and the value of –log10(P) on Y scale. LOF variants were displayed in triangles, while the remaining PA variants were displayed in circles, which contain the number of mutation carriers. Consequence types were denoted by different colors. Variant IDs for mutations which were nominally significant and had predicted consequences were displayed in the plot. B The volcano plot illustrates the effect sizes of PA variants on X scale and the value of –log10(P) in y scale. Shape, color, and text annotations were the same as those in Panel A.

Table S8 Associations between PA variants in the coding sequence of *G6PC* gene and serum CRP (natural-log transformed) in the meta-analysis set

| variant_id | reference_allele | alternate_allele | transcript | Consequense | CDS_position | Amino_acids | number_of_ref_allele_homozygote | number_of_heterozygote | number_of_alt_allele_homozygote | alt_allele_frequency | alt_allele_beta(se) | p_value | FDR_p_value |
| --- | --- | --- | --- | --- | --- | --- | --- | --- | --- | --- | --- | --- | --- |
| 17:42900952:D:1 | TC | T | ENST00000253801 | Frameshift variant | 77 | S/X | 120996 | 76 | 0 | 3.14e-04 | 0.326(0.12) | 6.50e-03 | 1.93e-01 |
| 17:42909416:C:G | C | G | ENST00000253801 |  | 560 | S/* | 121070 | 2 | 0 | 8.26e-06 | 1.96(0.738) | 7.92e-03 | 1.93e-01 |
| 17:42900890:T:G | T | G | ENST00000253801 | Missense variant | 14 | M/R | 121071 | 1 | 0 | 4.13e-06 | 2.696(1.044) | 9.81e-03 | 1.93e-01 |
| 17:42901069:G:T | G | T | ENST00000253801 | Missense variant | 193 | A/S | 121062 | 10 | 0 | 4.13e-05 | 0.724(0.33) | 2.82e-02 | 4.16e-01 |
| 17:42900985:G:T | G | T | ENST00000253801 | Missense variant | 109 | A/S | 121071 | 1 | 0 | 4.13e-06 | 2.144(1.044) | 4.00e-02 | 4.72e-01 |
| 17:42900985:G:A | G | A | ENST00000253801 | Missense variant | 109 | A/T | 121067 | 5 | 0 | 2.06e-05 | -0.918(0.467) | 4.93e-02 | 4.85e-01 |
| 17:42911403:C:T | C | T | ENST00000253801 | Stop gained | 1051 | Q/* | 121071 | 1 | 0 | 4.13e-06 | 1.8(1.044) | 8.46e-02 | 5.91e-01 |
| 17:42910935:T:C | T | C | ENST00000253801 | Missense variant | 583 | F/L | 121070 | 2 | 0 | 8.26e-06 | 1.255(0.738) | 8.91e-02 | 5.91e-01 |
| 17:42903947:C:T | C | T | ENST00000253801 | Missense variant | 247 | R/C | 121012 | 60 | 0 | 2.48e-04 | 0.228(0.135) | 9.04e-02 | 5.91e-01 |
| 17:42900931:C:T | C | T | ENST00000253801 | Missense variant | 55 | L/F | 121071 | 1 | 0 | 4.13e-06 | 1.712(1.044) | 1.01e-01 | 5.91e-01 |
| 17:42909418:G:C | G | C | ENST00000253801 | Missense variant | 562 | G/R | 121055 | 17 | 0 | 7.02e-05 | 0.394(0.253) | 1.20e-01 | 5.91e-01 |
| 17:42903992:D:1 | TC | T | ENST00000253801 | Frameshift variant | 293 | S/X | 121071 | 1 | 0 | 4.13e-06 | 1.606(1.044) | 1.24e-01 | 5.91e-01 |
| 17:42911368:G:A | G | A | ENST00000253801 | Missense variant | 1016 | S/N | 121071 | 1 | 0 | 4.13e-06 | 1.58(1.044) | 1.30e-01 | 5.91e-01 |
| 17:42909409:G:A | G | A | ENST00000253801 | Missense variant | 553 | V/I | 121067 | 5 | 0 | 2.07e-05 | -0.661(0.467) | 1.57e-01 | 6.43e-01 |
| 17:42911336:C:A | C | A | ENST00000253801 | Stop gained | 984 | C/* | 121071 | 1 | 0 | 4.13e-06 | 1.455(1.044) | 1.63e-01 | 6.43e-01 |
| 17:42910977:T:C | T | C | ENST00000253801 | Missense variant | 625 | Y/H | 121071 | 1 | 0 | 4.13e-06 | 1.401(1.044) | 1.80e-01 | 6.62e-01 |
| 17:42911391:C:T | C | T | ENST00000253801 | Stop gained | 1039 | Q/* | 120977 | 95 | 0 | 3.92e-04 | 0.136(0.107) | 2.05e-01 | 7.11e-01 |
| 17:42901018:C:G | C | G | ENST00000253801 | Missense variant | 142 | P/A | 121070 | 2 | 0 | 8.26e-06 | 0.904(0.738) | 2.21e-01 | 7.23e-01 |
| 17:42907538:A:G | A | G | ENST00000253801 | Missense variant | 356 | H/R | 121070 | 2 | 0 | 8.26e-06 | 0.821(0.738) | 2.66e-01 | 8.14e-01 |
| 17:42911190:T:A | T | A | ENST00000253801 | Missense variant | 838 | Y/N | 121071 | 1 | 0 | 4.13e-06 | 1.114(1.044) | 2.86e-01 | 8.14e-01 |
| 17:42903935:C:A | C | A | ENST00000253801 | Missense variant | 235 | L/I | 121071 | 1 | 0 | 4.13e-06 | -1.105(1.044) | 2.90e-01 | 8.14e-01 |
| 17:42901084:T:G | T | G | ENST00000253801 | Missense variant | 208 | W/G | 121070 | 2 | 0 | 8.26e-06 | 0.692(0.738) | 3.49e-01 | 9.32e-01 |
| 17:42900977:C:G | C | G | ENST00000253801 | Missense variant | 101 | S/C | 121070 | 2 | 0 | 8.26e-06 | -0.597(0.738) | 4.19e-01 | 9.32e-01 |
| 17:42910924:T:C | T | C | ENST00000253801 | Missense variant | 572 | V/A | 121071 | 1 | 0 | 4.13e-06 | -0.808(1.044) | 4.39e-01 | 9.32e-01 |
| 17:42900989:A:T | A | T | ENST00000253801 | Missense variant | 113 | D/V | 121059 | 13 | 0 | 5.37e-05 | 0.221(0.29) | 4.45e-01 | 9.32e-01 |
| 17:42910965:A:G | A | G | ENST00000253801 | Missense variant | 613 | S/G | 121070 | 2 | 0 | 8.26e-06 | 0.551(0.738) | 4.56e-01 | 9.32e-01 |
| 17:42907564:T:C | T | C | ENST00000253801 | Missense variant | 382 | Y/H | 121071 | 1 | 0 | 4.13e-06 | 0.729(1.044) | 4.85e-01 | 9.32e-01 |
| 17:42911124:T:C | T | C | ENST00000253801 | Missense variant | 772 | F/L | 121071 | 1 | 0 | 4.13e-06 | -0.715(1.044) | 4.94e-01 | 9.32e-01 |
| 17:42911029:T:C | T | C | ENST00000253801 | Missense variant | 677 | L/P | 121071 | 1 | 0 | 4.13e-06 | -0.671(1.044) | 5.20e-01 | 9.32e-01 |
| 17:42900910:G:C | G | C | ENST00000253801 | Missense variant | 34 | G/R | 121070 | 2 | 0 | 8.26e-06 | 0.436(0.738) | 5.55e-01 | 9.32e-01 |
| 17:42903948:G:A | G | A | ENST00000253801 | Missense variant | 248 | R/H | 121068 | 4 | 0 | 1.65e-05 | -0.298(0.522) | 5.68e-01 | 9.32e-01 |
| 17:42911419:D:3 | CGTT | C | ENST00000253801 | Inframe deletion | 1068-1070 | SL/S | 121068 | 4 | 0 | 1.65e-05 | 0.29(0.522) | 5.78e-01 | 9.32e-01 |
| 17:42911110:T:C | T | C | ENST00000253801 | Missense variant | 758 | I/T | 121070 | 2 | 0 | 8.26e-06 | 0.382(0.738) | 6.05e-01 | 9.32e-01 |
| 17:42901066:G:A | G | A | ENST00000253801 | Missense variant | 190 | V/I | 121071 | 1 | 0 | 4.13e-06 | 0.537(1.044) | 6.07e-01 | 9.32e-01 |
| 17:42911318:C:A | C | A | ENST00000253801 | Missense variant | 966 | F/L | 121051 | 21 | 0 | 8.67e-05 | 0.116(0.228) | 6.10e-01 | 9.32e-01 |
| 17:42907547:G:A | G | A | ENST00000253801 | Missense variant | 365 | G/D | 121071 | 1 | 0 | 4.13e-06 | 0.524(1.044) | 6.15e-01 | 9.32e-01 |
| 17:42911131:D:3 | GCCT | G | ENST00000253801 | Inframe deletion | 780-782 | SL/S | 121071 | 1 | 0 | 4.13e-06 | -0.524(1.044) | 6.16e-01 | 9.32e-01 |
| 17:42911321:C:A | C | A | ENST00000253801 | Stop gained | 969 | Y/* | 121056 | 16 | 0 | 6.61e-05 | 0.129(0.261) | 6.21e-01 | 9.32e-01 |
| 17:42904028:G:A | G | A | ENST00000253801 | Missense variant | 328 | E/K | 121071 | 1 | 0 | 4.13e-06 | 0.508(1.044) | 6.27e-01 | 9.32e-01 |
| 17:42910932:A:C | A | C | ENST00000253801 | Missense variant | 580 | T/P | 121070 | 2 | 0 | 8.26e-06 | -0.349(0.738) | 6.37e-01 | 9.32e-01 |
| 17:42909410:T:C | T | C | ENST00000253801 | Missense variant | 554 | V/A | 121070 | 2 | 0 | 8.26e-06 | -0.328(0.738) | 6.57e-01 | 9.32e-01 |
| 17:42907552:G:A | G | A | ENST00000253801 | Missense variant | 370 | A/T | 121066 | 6 | 0 | 2.48e-05 | -0.168(0.426) | 6.93e-01 | 9.32e-01 |
| 17:42907543:A:G | A | G | ENST00000253801 | Missense variant | 361 | M/V | 121071 | 1 | 0 | 4.13e-06 | -0.401(1.044) | 7.01e-01 | 9.32e-01 |
| 17:42911344:C:T | C | T | ENST00000253801 | Missense variant | 992 | A/V | 121038 | 34 | 0 | 1.40e-04 | -0.066(0.179) | 7.12e-01 | 9.32e-01 |
| 17:42911149:D:1 | GC | G | ENST00000253801 | Frameshift variant | 798 | G/X | 121071 | 1 | 0 | 4.13e-06 | 0.378(1.044) | 7.17e-01 | 9.32e-01 |
| 17:42909364:C:T | C | T | ENST00000253801 | Stop gained | 508 | R/* | 121061 | 11 | 0 | 4.54e-05 | -0.105(0.315) | 7.38e-01 | 9.32e-01 |
| 17:42911235:C:T | C | T | ENST00000253801 | Missense variant | 883 | R/C | 121065 | 7 | 0 | 2.89e-05 | 0.13(0.395) | 7.42e-01 | 9.32e-01 |
| 17:42911326:T:C | T | C | ENST00000253801 | Missense variant | 974 | L/S | 121067 | 5 | 0 | 2.06e-05 | 0.132(0.467) | 7.78e-01 | 9.37e-01 |
| 17:42911161:G:T | G | T | ENST00000253801 | Missense variant | 809 | G/V | 121071 | 1 | 0 | 4.13e-06 | 0.293(1.044) | 7.79e-01 | 9.37e-01 |
| 17:42909403:D:1 | GC | G | ENST00000253801 | Frameshift variant | 548 | A/X | 121071 | 1 | 0 | 4.13e-06 | -0.211(1.044) | 8.40e-01 | 9.37e-01 |
| 17:42911127:G:A | G | A | ENST00000253801 | Missense variant | 775 | A/T | 121071 | 1 | 0 | 4.13e-06 | -0.205(1.044) | 8.44e-01 | 9.37e-01 |
| 17:42911295:C:G | C | G | ENST00000253801 | Missense variant | 943 | P/A | 121029 | 43 | 0 | 1.78e-04 | -0.029(0.159) | 8.57e-01 | 9.37e-01 |
| 17:42910937:C:G | C | G | ENST00000253801 | Missense variant | 585 | F/L | 121070 | 2 | 0 | 8.26e-06 | -0.121(0.738) | 8.70e-01 | 9.37e-01 |
| 17:42901013:T:C | T | C | ENST00000253801 | Missense variant | 137 | L/P | 121071 | 1 | 0 | 4.13e-06 | -0.169(1.044) | 8.71e-01 | 9.37e-01 |
| 17:42907567:G:A | G | A | ENST00000253801 | Missense variant | 385 | V/M | 121071 | 1 | 0 | 4.13e-06 | -0.166(1.044) | 8.74e-01 | 9.37e-01 |
| 17:42901067:T:C | T | C | ENST00000253801 | Missense variant | 191 | V/A | 121071 | 1 | 0 | 4.13e-06 | 0.085(1.044) | 9.35e-01 | 9.67e-01 |
| 17:42909323:G:T | G | T | ENST00000253801 | Missense variant | 467 | W/L | 121071 | 1 | 0 | 4.13e-06 | 0.079(1.044) | 9.40e-01 | 9.67e-01 |
| 17:42900878:T:A | T | A | ENST00000253801 | Start lost | 2 | M/K | 121071 | 1 | 0 | 4.13e-06 | 0.062(1.044) | 9.53e-01 | 9.67e-01 |
| 17:42909418:G:A | G | A | ENST00000253801 | Missense variant | 562 | G/S | 121056 | 16 | 0 | 6.61e-05 | 0.011(0.261) | 9.67e-01 | 9.67e-01 |
| 17:42901065:G:A | G | A | ENST00000253801 | Stop gained | 189 | W/* | 121072 | 0 | 0 | 0.00e+00 |  |  | NA |
| 17:42903939:T:A | T | A | ENST00000253801 | Missense variant | 239 | F/Y | 121072 | 0 | 0 | 0.00e+00 |  |  | NA |
| 17:42903950:C:T | C | T | ENST00000253801 | Missense variant | 250 | P/S | 121072 | 0 | 0 | 0.00e+00 |  |  | NA |
| 17:42903958:G:A | G | A | ENST00000253801 | Stop gained | 258 | W/* | 121072 | 0 | 0 | 0.00e+00 |  |  | NA |
| 17:42903968:G:A | G | A | ENST00000253801 | Missense variant | 268 | D/N | 121072 | 0 | 0 | 0.00e+00 |  |  | NA |
| 17:42907523:G:A | G | A | ENST00000253801 | Missense variant | 341 | G/E | 121072 | 0 | 0 | 0.00e+00 |  |  | NA |
| 17:42907545:G:A | G | A | ENST00000253801 | Missense variant | 363 | M/I | 121072 | 0 | 0 | 0.00e+00 |  |  | NA |
| 17:42907563:C:G | C | G | ENST00000253801 | Stop gained | 381 | Y/* | 121072 | 0 | 0 | 0.00e+00 |  |  | NA |
| 17:42907577:C:T | C | T | ENST00000253801 | Missense variant | 395 | T/I | 121072 | 0 | 0 | 0.00e+00 |  |  | NA |
| 17:42907582:A:C | A | C | ENST00000253801 | Missense variant | 400 | T/P | 121072 | 0 | 0 | 0.00e+00 |  |  | NA |
| 17:42909365:G:A | G | A | ENST00000253801 | Missense variant | 509 | R/Q | 121072 | 0 | 0 | 0.00e+00 |  |  | NA |
| 17:42910962:G:T | G | T | ENST00000253801 | Missense variant | 610 | A/S | 121072 | 0 | 0 | 0.00e+00 |  |  | NA |
| 17:42911074:C:T | C | T | ENST00000253801 | Missense variant | 722 | A/V | 121072 | 0 | 0 | 0.00e+00 |  |  | NA |
| 17:42911076:C:T | C | T | ENST00000253801 | Stop gained | 724 | Q/* | 121072 | 0 | 0 | 0.00e+00 |  |  | NA |
| 17:42911296:C:T | C | T | ENST00000253801 | Missense variant | 944 | P/L | 121072 | 0 | 0 | 0.00e+00 |  |  | NA |
| 17:42911336:C:G | C | G | ENST00000253801 | Missense variant | 984 | C/W | 121072 | 0 | 0 | 0.00e+00 |  |  | NA |
| 17:42911355:D:1 | CT | C | ENST00000253801 | Frameshift variant | 1004 | L/X | 121072 | 0 | 0 | 0.00e+00 |  |  | NA |

Table S9 Leave-one-variant-out analysis between PA mutation burden in *CRP* gene and serum CRP (natural log transformed)

| Variant being excluded | Number of PA allele carriers | Effect size | Standard error | P value |
| --- | --- | --- | --- | --- |
| 1:159713525:T:A | 312 | -0.676 | 0.059 | 3.10e-30 |
| 1:159713531:C:T | 305 | -0.666 | 0.06 | 1.04e-28 |
| 1:159713542:G:A | 313 | -0.676 | 0.059 | 2.71e-30 |
| 1:159713560:C:T | 312 | -0.679 | 0.059 | 2.09e-30 |
| 1:159713583:C:G | 303 | -0.664 | 0.06 | 2.16e-28 |
| 1:159713584:G:A | 312 | -0.676 | 0.059 | 3.49e-30 |
| 1:159713624:G:C | 310 | -0.689 | 0.059 | 4.01e-31 |
| 1:159713660:C:T | 312 | -0.675 | 0.059 | 4.21e-30 |
| 1:159713663:C:T | 312 | -0.676 | 0.059 | 3.12e-30 |
| 1:159713663:C:A | 312 | -0.672 | 0.059 | 7.61e-30 |
| 1:159713664:A:G | 310 | -0.672 | 0.059 | 1.10e-29 |
| 1:159713673:T:A | 312 | -0.678 | 0.059 | 2.21e-30 |
| 1:159713679:A:G | 312 | -0.675 | 0.059 | 4.35e-30 |
| 1:159713719:C:T | 284 | -0.676 | 0.062 | 1.17e-27 |
| Ü1:159713728:C:A | 312 | -0.679 | 0.059 | 1.93e-30 |
| 1:159713733:T:C | 312 | -0.671 | 0.059 | 8.77e-30 |
| 1:159713739:C:T | 312 | -0.678 | 0.059 | 2.13e-30 |
| 1:159713764:D:2 | 312 | -0.674 | 0.059 | 4.68e-30 |
| 1:159713778:D:9 | 312 | -0.674 | 0.059 | 4.92e-30 |
| 1:159713790:T:G | 313 | -0.676 | 0.059 | 2.71e-30 |
| 1:159713791:T:A | 313 | -0.676 | 0.059 | 2.71e-30 |
| 1:159713803:I:4 | 311 | -0.68 | 0.059 | 2.05e-30 |
| 1:159713822:C:A | 311 | -0.671 | 0.059 | 1.10e-29 |
| 1:159713827:C:T | 311 | -0.677 | 0.059 | 3.37e-30 |
| 1:159713830:T:C | 313 | -0.676 | 0.059 | 2.71e-30 |
| 1:159713863:G:A | 311 | -0.677 | 0.059 | 3.37e-30 |
| 1:159713869:G:C | 311 | -0.669 | 0.059 | 1.48e-29 |
| 1:159713874:I:1 | 312 | -0.677 | 0.059 | 2.80e-30 |
| 1:159713896:A:G | 300 | -0.678 | 0.06 | 2.70e-29 |
| 1:159713912:D:1 | 312 | -0.677 | 0.059 | 2.79e-30 |
| 1:159713950:A:T | 312 | -0.675 | 0.059 | 4.05e-30 |
| 1:159713952:A:G | 312 | -0.678 | 0.059 | 2.50e-30 |
| 1:159713967:T:C | 313 | -0.676 | 0.059 | 2.71e-30 |
| 1:159713972:I:1 | 312 | -0.674 | 0.059 | 5.05e-30 |
| 1:159713975:C:A | 310 | -0.677 | 0.059 | 4.11e-30 |
| 1:159713988:G:A | 306 | -0.672 | 0.06 | 2.78e-29 |
| 1:159713991:A:G | 311 | -0.678 | 0.059 | 2.93e-30 |
| 1:159714006:C:T | 309 | -0.68 | 0.059 | 2.72e-30 |
| 1:159714007:G:A | 295 | -0.652 | 0.061 | 9.92e-27 |
| 1:159714025:T:C | 307 | -0.694 | 0.06 | 2.72e-31 |
| 1:159714026:G:C | 250 | -0.686 | 0.066 | 3.03e-25 |
| 1:159714066:G:A | 285 | -0.702 | 0.062 | 9.20e-30 |
| 1:159714081:A:G | 312 | -0.679 | 0.059 | 2.04e-30 |
| 1:159714089:A:T | 312 | -0.678 | 0.059 | 2.41e-30 |
| 1:159714102:G:A | 312 | -0.675 | 0.059 | 4.40e-30 |
| 1:159714105:T:C | 311 | -0.678 | 0.059 | 2.93e-30 |
| 1:159714112:G:C | 312 | -0.674 | 0.059 | 4.88e-30 |
| 1:159714124:C:A | 311 | -0.677 | 0.059 | 3.40e-30 |
| 1:159714136:T:C | 312 | -0.672 | 0.059 | 6.79e-30 |
| 1:159714463:A:G | 245 | -0.685 | 0.067 | 1.09e-24 |
| 1:159714473:A:C | 311 | -0.674 | 0.059 | 6.06e-30 |
| 1:159714484:A:G | 306 | -0.663 | 0.06 | 1.44e-28 |

Table S10 Leave-one-variant-out analysis between PA mutation burden in *G6PC* gene and serum CRP (natural log transformed)

| Variant being excluded | Number of PA allele carriers | Effect size | Standard error | P value |
| --- | --- | --- | --- | --- |
| 17:42900878:T:A | 661 | 0.172 | 0.041 | 2.30e-05 |
| 17:42900890:T:G | 662 | 0.168 | 0.041 | 3.47e-05 |
| 17:42900910:G:C | 661 | 0.172 | 0.041 | 2.54e-05 |
| 17:42900931:C:T | 661 | 0.17 | 0.041 | 2.97e-05 |
| 17:42900952:D:1 | 574 | 0.155 | 0.044 | 3.90e-04 |
| 17:42900977:C:G | 661 | 0.175 | 0.041 | 1.80e-05 |
| 17:42900985:G:A | 657 | 0.182 | 0.041 | 8.14e-06 |
| 17:42900985:G:T | 661 | 0.171 | 0.041 | 2.63e-05 |
| 17:42900989:A:T | 645 | 0.175 | 0.041 | 2.22e-05 |
| 17:42901013:T:C | 662 | 0.173 | 0.041 | 2.17e-05 |
| 17:42901018:C:G | 661 | 0.17 | 0.041 | 2.97e-05 |
| 17:42901065:G:A | 662 | 0.171 | 0.041 | 2.78e-05 |
| 17:42901066:G:A | 662 | 0.172 | 0.041 | 2.44e-05 |
| 17:42901067:T:C | 662 | 0.172 | 0.041 | 2.26e-05 |
| 17:42901069:G:T | 647 | 0.158 | 0.041 | 1.28e-04 |
| 17:42901084:T:G | 658 | 0.168 | 0.041 | 4.01e-05 |
| 17:42903935:C:A | 662 | 0.174 | 0.041 | 1.86e-05 |
| 17:42903939:T:A | 661 | 0.175 | 0.041 | 1.72e-05 |
| 17:42903947:C:T | 582 | 0.167 | 0.043 | 1.23e-04 |
| 17:42903948:G:A | 657 | 0.171 | 0.041 | 2.73e-05 |
| 17:42903950:C:T | 663 | 0.172 | 0.041 | 2.26e-05 |
| 17:42903958:G:A | 662 | 0.172 | 0.041 | 2.38e-05 |
| 17:42903968:G:A | 663 | 0.172 | 0.041 | 2.26e-05 |
| 17:42903992:D:1 | 662 | 0.17 | 0.041 | 2.91e-05 |
| 17:42904028:G:A | 662 | 0.172 | 0.041 | 2.43e-05 |
| 17:42907523:G:A | 662 | 0.171 | 0.041 | 2.66e-05 |
| 17:42907538:A:G | 661 | 0.17 | 0.041 | 2.88e-05 |
| 17:42907543:A:G | 662 | 0.173 | 0.041 | 2.08e-05 |
| 17:42907545:G:A | 662 | 0.173 | 0.041 | 2.17e-05 |
| 17:42907547:G:A | 662 | 0.172 | 0.041 | 2.43e-05 |
| 17:42907552:G:A | 651 | 0.17 | 0.041 | 3.26e-05 |
| 17:42907563:C:G | 661 | 0.176 | 0.041 | 1.52e-05 |
| 17:42907564:T:C | 662 | 0.171 | 0.041 | 2.51e-05 |
| 17:42907567:G:A | 662 | 0.173 | 0.041 | 2.17e-05 |
| 17:42907577:C:T | 663 | 0.172 | 0.041 | 2.26e-05 |
| 17:42907582:A:C | 662 | 0.174 | 0.041 | 1.96e-05 |
| 17:42909323:G:T | 662 | 0.172 | 0.041 | 2.26e-05 |
| 17:42909364:C:T | 647 | 0.175 | 0.041 | 2.15e-05 |
| 17:42909365:G:A | 663 | 0.172 | 0.041 | 2.26e-05 |
| 17:42909403:D:1 | 662 | 0.173 | 0.041 | 2.15e-05 |
| 17:42909409:G:A | 658 | 0.179 | 0.041 | 1.20e-05 |
| 17:42909410:T:C | 661 | 0.174 | 0.041 | 1.97e-05 |
| 17:42909416:C:G | 661 | 0.167 | 0.041 | 4.17e-05 |
| 17:42909418:G:A | 641 | 0.18 | 0.041 | 1.42e-05 |
| 17:42909418:G:C | 643 | 0.167 | 0.041 | 5.48e-05 |
| 17:42910924:T:C | 662 | 0.174 | 0.041 | 1.95e-05 |
| 17:42910932:A:C | 660 | 0.172 | 0.041 | 2.33e-05 |
| 17:42910935:T:C | 661 | 0.169 | 0.041 | 3.32e-05 |
| 17:42910937:C:G | 660 | 0.173 | 0.041 | 2.16e-05 |
| 17:42910962:G:T | 661 | 0.169 | 0.041 | 3.33e-05 |
| 17:42910965:A:G | 661 | 0.171 | 0.041 | 2.64e-05 |
| 17:42910977:T:C | 662 | 0.17 | 0.041 | 2.81e-05 |
| 17:42911029:T:C | 662 | 0.174 | 0.041 | 1.99e-05 |
| 17:42911074:C:T | 662 | 0.171 | 0.041 | 2.60e-05 |
| 17:42911076:C:T | 663 | 0.172 | 0.041 | 2.26e-05 |
| 17:42911110:T:C | 661 | 0.172 | 0.041 | 2.49e-05 |
| 17:42911124:T:C | 661 | 0.175 | 0.041 | 1.73e-05 |
| 17:42911127:G:A | 661 | 0.173 | 0.041 | 2.12e-05 |
| 17:42911131:D:3 | 662 | 0.173 | 0.041 | 2.04e-05 |
| 17:42911149:D:1 | 662 | 0.172 | 0.041 | 2.37e-05 |
| 17:42911161:G:T | 661 | 0.172 | 0.041 | 2.35e-05 |
| 17:42911190:T:A | 662 | 0.171 | 0.041 | 2.68e-05 |
| 17:42911235:C:T | 653 | 0.173 | 0.041 | 2.31e-05 |
| 17:42911295:C:G | 606 | 0.188 | 0.043 | 9.57e-06 |
| 17:42911296:C:T | 662 | 0.171 | 0.041 | 2.60e-05 |
| 17:42911318:C:A | 638 | 0.175 | 0.041 | 2.40e-05 |
| 17:42911321:C:A | 638 | 0.184 | 0.041 | 9.45e-06 |
| 17:42911326:T:C | 655 | 0.173 | 0.041 | 2.32e-05 |
| 17:42911336:C:G | 662 | 0.173 | 0.041 | 2.06e-05 |
| 17:42911336:C:A | 662 | 0.17 | 0.041 | 2.83e-05 |
| 17:42911344:C:T | 620 | 0.184 | 0.042 | 1.17e-05 |
| 17:42911355:D:1 | 662 | 0.173 | 0.041 | 2.22e-05 |
| 17:42911368:G:A | 662 | 0.17 | 0.041 | 2.89e-05 |
| 17:42911391:C:T | 542 | 0.17 | 0.045 | 1.56e-04 |
| 17:42911403:C:T | 662 | 0.17 | 0.041 | 3.00e-05 |
| 17:42911419:D:3 | 658 | 0.171 | 0.041 | 2.72e-05 |

Table S11 Baseline characteristics of study population by PA mutation burden in the *CRP* gene

|  | Carriers of PA alleles in the *CRP* gene | Non-carriers | P for group difference |
| --- | --- | --- | --- |
| N | 313 | 161117 |  |
| Age (year, mean, SD) | 56.9 (7.6) | 56.7 (8.0) | 0.58 |
| Men (N, proportion) | 128 (40.9%) | 72,637 (45.1%) | 0.14 |
| High physical activity (N, proportion) | 147 (47.4%) | 77,925 (48.5%) | 0.73 |
| Ever-smoker (N, proportion) | 189 (60.6%) | 98,999 (61.7%) | 0.73 |
| Daily alcohol use (N, proportion) | 62 (19.8%) | 34,197 (21.2%) | 0.58 |
| BMI (kg/m^^2^, mean, SD) | 27.4 (4.8) | 27.3 (4.7) | 0.66 |
| Waist circumference (cm, mean, SD) | 89.6 (13.3) | 90.0 (13.4) | 0.62 |
| Systolic blood pressure (mmHg, mean, SD) | 137.6 (17.9) | 137.8 (18.5) | 0.82 |
| Serum biomarkers |  |  |  |
| Glucose (mmol/L) | 5.09 (0.84) | 5.11 (1.18) | 0.67 |
| Triglycerides (mmol/L) | 1.50 (1.07) | 1.48 (1.09) | 0.47 |
| HDL cholesterol (mmol/L) | 1.49 (0.40) | 1.46 (0.38) | 0.28 |
| CRP (mg/L) | 0.66 (1.09) | 1.30 (2.04) | 3.94e-13 |
| Natural logarithm CRP | -0.35 (1.09) | 0.31 (1.05) | 7.08e-23 |

^1^ P for group difference between carriers and non-carriers, estimated by Fisher's exact test, t-test, and non-parametric median test whenever appropriate.

Table S12 Baseline characteristics of study population by PA mutation burden in the *G6PC* gene

|  | Carriers of PA alleles in the *G6PC* gene | Non-carriers | P for group difference |
| --- | --- | --- | --- |
| N | 663 | 160767 |  |
| Age (year, mean, SD) | 57.4 (7.7) | 56.7 (8.0) | 0.01 |
| Men (N, proportion) | 298 (44.9%) | 72,467 (45.1%) | 0.97 |
| In first 50k release (N, proportion) | 172 (25.9%) | 39,077 (24.3%) | 0.34 |
| High physical activity (N, proportion) | 328 (49.8%) | 77,744 (48.5%) | 0.51 |
| Ever-smoker (N, proportion) | 411 (62.0%) | 98,777 (61.7%) | 0.87 |
| Daily alcohol use (N, proportion) | 122 (18.4%) | 34,137 (21.2%) | 0.08 |
| BMI (kg/m^^2^, mean, SD) | 26.9 (4.8) | 27.3 (4.7) | 0.03 |
| Waist circumference (cm, mean, SD) | 88.7 (13.3) | 90.0 (13.4) | 0.01 |
| Body fat percentage (%, mean, SD) | 31.0 (8.4) | 31.4 (8.5) | 0.26 |
| Systolic blood pressure (mmHg, mean, SD) | 139.3 (18.4) | 137.8 (18.5) | 0.04 |
| Serum biomarkers |  |  |  |
| Glucose (mmol/L, mean, SD) | 5.03 (1.02) | 5.11 (1.18) | 0.04 |
| Triglycerides (mmol/L, mean, SD) | 1.66 (1.20) | 1.48 (1.09) | 3.34e-05 |
| HDL cholesterol (mmol/L, mean, SD) | 1.48 (0.39) | 1.46 (0.38) | 0.24 |
| CRP (mg/L, median, IQR) | 1.60 (2.33) | 1.30 (2.03) | 4.92e-05 |
| Natural logarithm CRP (mean,SD) | 0.49 (1.03) | 0.31 (1.06) | 7.73e-06 |

^1^ P for group difference between carriers and non-carriers, estimated by Fisher's exact test, t-test, and non-parametric median test whenever appropriate.

Table S13 Serum CRP (natural log transformed) by PA mutation burden, BMI category, and waist circumference category

|  | Number of participants (proportion) | Natural logarithm of CRP (mean[SD]) | CRP (median[IQR]) |
| --- | --- | --- | --- |
| All participants | 161,430(100%) | 0.31(1.06) | 1.3(2.03) |
| PA mutation burden in the *CRP* gene |  |  |  |
| non-PA carriers | 161,117(99.8%) | 0.31(1.05) | 1.30(2.04) |
| PA carriers | 313(0.2%) | -0.35(1.09) | 0.66(1.09) |
| PA mutation burden in the *G6PC* gene |  |  |  |
| non-PA carriers | 160,767(99.6%) | 0.31(1.06) | 1.30(2.03) |
| PA carriers | 663(0.4%) | 0.49(1.03) | 1.60(2.33) |
| BMI categories |  |  |  |
| Underweight | 769(0.5%) | -0.63(1.15) | 0.41(0.70) |
| Normal | 53,589(33.2%) | -0.17(1.01) | 0.77(1.12) |
| Overweight | 68,551(42.5%) | 0.34(0.95) | 1.33(1.77) |
| Obese | 38,009(23.5%) | 0.95(0.93) | 2.52(3.32) |
| Missing value | 512(0.3%) | 0.76(1.13) | 2.08(3.26) |
| Waist circumference |  |  |  |
| Normal | 64,256(39.8%) | -0.13(1.00) | 0.80(1.15) |
| Moderate central fat accumulation | 43,793(27.1%) | 0.32(0.94) | 1.31(1.73) |
| High central fat accumulation | 53,076(32.9%) | 0.84(0.95) | 2.25(3.05) |
| Missing value | 305(0.2%) | 0.70(1.19) | 1.98(3.23) |

Table S14 Effect of PA mutation burden, BMI category, and waist circumference category on serum CRP (natural log transformed)

|  | Coefficient (95% CI) | P value |
| --- | --- | --- |
| PA mutation burden in the *CRP* gene |  |  |
| non-PA carriers | Reference |  |
| PA carriers | -0.68 (-0.79, -0.56) | 2.26e-30 |
| PA mutation burden in the *G6PC* gene |  |  |
| non-PA carriers | Reference |  |
| PA carriers | 0.17 (0.09, 0.25) | 1.94e-05 |
| BMI categories |  |  |
| Normal | Reference |  |
| Underweight | -0.49 (-0.56, -0.42) | 7.05e-45 |
| Overweight | 0.52 ( 0.50, 0.53) | <1e-100 |
| Obese | 1.12 ( 1.10, 1.13) | <1e-100 |
| Waist circumference |  |  |
| Normal | Reference |  |
| Moderate central fat accumulation | 0.44 ( 0.43, 0.45) | <1e-100 |
| High central fat accumulation | 0.94 ( 0.93, 0.95) | <1e-100 |

Table S15 Interaction effect of PA mutation burden in the *CRP* gene with BMI category, and waist circumference category on serum CRP (natural log transformed)

|  | Number of individuals | Mean (SD) of serum CRP (natural log transformed) | Beta coefficient (95% CI) in the stratified models | P for beta coefficient in the stratified models | P for interaction terms in the interaction models |
| --- | --- | --- | --- | --- | --- |
| Obesity status (measured by BMI) | | | | | |
| Non-carriers |  |  |  |  |  |
| Normal | 53,485 | -0.16 (1.01) | Reference |  |  |
| Overweight | 68,426 | 0.34 (0.95) | 0.52 (0.50, 0.53) | <1e-100 |  |
| Obese | 37,927 | 0.95 (0.93) | 1.12 (1.10, 1.13) | <1e-100 |  |
| PA mutation carriers |  |  |  |  |  |
| Normal | 104 | -0.74 (1.21) | Reference |  |  |
| Overweight | 125 | -0.24 (0.95) | 0.53 (0.25, 0.81) | 3.00e-04 | 0.913 |
| Obese | 82 | -0.01 (0.98) | 0.75 (0.44, 1.07) | 4.82e-06 | 0.008 |
| Central obesity status (measured by waist circumference) | | | | | |
| Non-carriers |  |  |  |  |  |
| Normal | 64,141 | -0.13 (1.00) | Reference |  |  |
| Moderate central fat accumulation | 43,708 | 0.32 (0.94) | 0.44 (0.43, 0.45) | <1e-100 |  |
| High central fat accumulation | 52,964 | 0.84 (0.95) | 0.94 (0.93, 0.95) | <1e-100 |  |
| PA mutation carriers |  |  |  |  |  |
| Normal | 115 | -0.81 (1.05) | Reference |  |  |
| Moderate central fat accumulation | 85 | -0.18 (1.11) | 0.68 (0.39, 0.98) | 9.64e-06 | 0.164 |
| High central fat accumulation | 112 | 0.00 (0.96) | 0.82 (0.54, 1.10) | 2.50e-08 | 0.220 |

Table S16 Interaction effect of PA mutation burden in the *G6PC* gene with BMI category, and waist circumference category on serum CRP (natural log transformed)

|  | Number of individuals | Mean (SD) of serum CRP (natural log transformed) | Beta coefficient (95% CI) in the stratified models | P for beta coefficient in the stratified models | P for interaction terms in the interaction models |
| --- | --- | --- | --- | --- | --- |
| Obesity status (measured by BMI) | | | | | |
| Non-carriers |  |  |  |  |  |
| Normal | 53342 | -0.17 (1.01) | Reference |  |  |
| Overweight | 68284 | 0.34 (0.95) | 0.52 (0.50, 0.53) | <1e-100 |  |
| Obese | 37865 | 0.95 (0.93) | 1.12 (1.10, 1.13) | <1e-100 |  |
| PA mutation carriers |  |  |  |  |  |
| Normal | 247 | 0.09 (0.97) | Reference |  |  |
| Overweight | 267 | 0.56 (1.03) | 0.52 (0.36, 0.69) | 1.92e-09 | 0.813 |
| Obese | 144 | 1.07 (0.82) | 1.02 (0.82, 1.22) | 3.15e-22 | 0.223 |
| Central obesity status (measured by waist circumference) | | | | | |
| Non-carriers |  |  |  |  |  |
| Normal | 63960 | -0.13 (1.00) | Reference |  |  |
| Moderate central fat accumulation | 43630 | 0.32 (0.94) | 0.44 (0.43, 0.45) | <1e-100 |  |
| High central fat accumulation | 52874 | 0.84 (0.95) | 0.94 (0.93, 0.95) | <1e-100 |  |
| PA mutation carriers |  |  |  |  |  |
| Normal | 296 | 0.16 (0.98) | Reference |  |  |
| Moderate central fat accumulation | 163 | 0.53 (1.01) | 0.36 (0.16, 0.55) | 2.76e-04 | 0.389 |
| High central fat accumulation | 202 | 0.93 (0.94) | 0.76 (0.58, 0.93) | 2.03e-16 | 0.034 |

Table S17 Associations between PA mutation burden in the *CRP* gene and health outcomes

| Outcome | Cases among non-PA carriers | Cases among PA carriers | Odds ratio (95%CI) | P value | FDR-corrected P |
| --- | --- | --- | --- | --- | --- |
| Autoimmune/inflammatory |  |  |  |  |  |
| Celiac disease | 1043(0.6%) | 1(0.3%) | 0.06(0.00,65.02) | 0.434 | 0.977 |
| IBD (all types) | 9628(6%) | 17(5.4%) | 0.60(0.22,1.68) | 0.334 | 0.977 |
| Crohn’s disease | 930(0.6%) | 5(1.6%) | 1.35(0.14,13.15) | 0.799 | 0.991 |
| Ulcerative colitis | 1835(1.1%) | 5(1.6%) | 2.83(0.84,9.59) | 0.095 | 0.608 |
| Psoriatic arthritis | 472(0.3%) | 1(0.3%) | 0.00(0.00,1.99e+226) | 0.968 | 0.991 |
| Rheumatoid arthritis | 3628(2.3%) | 3(1%) | 0.51(0.07,3.66) | 0.501 | 0.991 |
| Type 1 diabetes | 1425(0.9%) | 2(0.6%) | 0.83(0.09,7.64) | 0.869 | 0.991 |
| Knee osteoarthritis | 12136(7.5%) | 24(7.7%) | 0.87(0.42,1.80) | 0.708 | 0.991 |
| Cardiovascular |  |  |  |  |  |
| Coronary artery disease | 14651(9.1%) | 27(8.6%) | 1.00(0.55,1.82) | 0.991 | 0.991 |
| Ischemic stroke (all type) | 2557(1.6%) | 3(1%) | 0.56(0.13,2.38) | 0.433 | 0.977 |
| Metabolic |  |  |  |  |  |
| Type 2 diabetes | 11238(7%) | 21(6.7%) | 1.05(0.53,2.07) | 0.885 | 0.991 |
| Chronic kidney disease | 5690(3.5%) | 16(5.1%) | 1.57(0.87,2.82) | 0.135 | 0.608 |
| Neurodegenerative |  |  |  |  |  |
| Alzheimer disease | 897(0.6%) | 2(0.6%) | 1.35(0.33,5.51) | 0.68 | 0.991 |
| Parkinson disease | 1082(0.7%) | 3(1%) | 1.80(0.47,6.92) | 0.392 | 0.977 |
| Psychiatric |  |  |  |  |  |
| Bipolar disorder | 741(0.5%) | 5(1.6%) | 4.35(0.72,26.32) | 0.109 | 0.608 |
| Depressive disorder | 14854(9.2%) | 33(10.6%) | 1.68(0.90,3.12) | 0.104 | 0.608 |

Table S18 Associations between PA mutation burden in the *G6PC* gene and health outcomes

| Outcome | Cases among non-PA carriers | Cases among PA carriers | Odds ratio (95%CI) | P value | FDR-corrected P |
| --- | --- | --- | --- | --- | --- |
| Autoimmune/inflammatory |  |  |  |  |  |
| Celiac disease | 1040(0.6%) | 4(0.6%) | 0.80(0.16,3.88) | 0.779 | 0.897 |
| IBD (all types) | 9604(6%) | 41(6.2%) | 1.29(0.71,2.34) | 0.396 | 0.897 |
| Crohn’s disease | 932(0.6%) | 3(0.5%) | 0.36(0.03,5.07) | 0.451 | 0.897 |
| Ulcerative colitis | 1838(1.1%) | 2(0.3%) | 0.55(0.08,3.96) | 0.554 | 0.897 |
| Psoriatic arthritis | 472(0.3%) | 1(0.2%) | 1.24(0.17,8.93) | 0.833 | 0.897 |
| Rheumatoid arthritis | 3613(2.2%) | 18(2.7%) | 2.00(1.03,3.89) | 0.04 | 0.486 |
| Type 1 diabetes | 1423(0.9%) | 4(0.6%) | 1.22(0.31,4.70) | 0.778 | 0.897 |
| Knee osteoarthritis | 12115(7.5%) | 45(6.8%) | 1.11(0.72,1.71) | 0.638 | 0.897 |
| Cardiovascular |  |  |  |  |  |
| Coronary artery disease | 14598(9.1%) | 80(12.1%) | 1.42(0.99,2.04) | 0.056 | 0.486 |
| Ischemic stroke (all type) | 2543(1.6%) | 17(2.6%) | 1.71(0.94,3.11) | 0.081 | 0.486 |
| Metabolic |  |  |  |  |  |
| Type 2 diabetes | 11212(7%) | 47(7.1%) | 1.06(0.68,1.65) | 0.797 | 0.897 |
| Chronic kidney disease | 5682(3.5%) | 24(3.6%) | 0.94(0.58,1.53) | 0.805 | 0.897 |
| Neurodegenerative |  |  |  |  |  |
| Alzheimer disease | 897(0.6%) | 2(0.3%) | 0.60(0.15,2.44) | 0.477 | 0.897 |
| Parkinson disease | 1078(0.7%) | 7(1.1%) | 1.96(0.84,4.57) | 0.117 | 0.527 |
| Psychiatric |  |  |  |  |  |
| Bipolar disorder | 741(0.5%) | 5(0.8%) | 1.22(0.16,9.48) | 0.847 | 0.897 |
| Depressive disorder | 14819(9.2%) | 68(10.3%) | 1.35(0.83,2.21) | 0.226 | 0.814 |
